# A Programmable Ontology Encompassing the Functional Logic of the *Drosophila* Brain

**DOI:** 10.3389/fninf.2022.853098

**Published:** 2022-06-20

**Authors:** Aurel A. Lazar, Mehmet Kerem Turkcan, Yiyin Zhou

**Affiliations:** Department of Electrical Engineering, Columbia University, New York, NY, United States

**Keywords:** *Drosophila melanogaster*, ontology, connectome/synaptome, feedback loops, functional logic, early olfactory system, *in silico* execution, cell type

## Abstract

The *Drosophila* brain has only a fraction of the number of neurons of higher organisms such as mice and humans. Yet the sheer complexity of its neural circuits recently revealed by large connectomics datasets suggests that computationally modeling the function of fruit fly brain circuits at this scale poses significant challenges. To address these challenges, we present here a programmable ontology that expands the scope of the current *Drosophila* brain anatomy ontologies to encompass the functional logic of the fly brain. The programmable ontology provides a language not only for modeling circuit motifs but also for programmatically exploring their functional logic. To achieve this goal, we tightly integrated the programmable ontology with the workflow of the interactive FlyBrainLab computing platform. As part of the programmable ontology, we developed NeuroNLP++, a web application that supports free-form English queries for constructing functional brain circuits fully anchored on the available connectome/synaptome datasets, and the published worldwide literature. In addition, we present a methodology for including a model of the space of odorants into the programmable ontology, and for modeling olfactory sensory circuits of the antenna of the fruit fly brain that detect odorant sources. Furthermore, we describe a methodology for modeling the functional logic of the antennal lobe circuit consisting of a massive number of local feedback loops, a characteristic feature observed across *Drosophila* brain regions. Finally, using a circuit library, we demonstrate the power of our methodology for interactively exploring the functional logic of the massive number of feedback loops in the antennal lobe.

## 1. Introduction

### 1.1. Challenges in Discovering the Functional Logic of Brain Circuits in the Connectomic/Synaptomic Era

Large scale foundational surveys of the anatomical, physiological and genomic architecture of brains of mice, primates and humans have shown the enormous variety of cell types (Tasic et al., 2018; Grünert and Martin, 2020; Bakken et al., 2021), diverse connectivity patterns with fan-ins and fan-outs in the tens of thousands and extensive feedback that vary both within and between brain regions (Harris et al., 2019). The last decade also saw an exponential growth in neuroscience data gathering, collection and availability, starting with the cubic millimeter brain tissue in mice and humans (Shapson-Coe et al., 2021). However, due to the sheer magnitude and complexity of brains of higher organisms, even with such data at hand, we are far behind in our understanding of the principles of neural computation in the brain.

Prior studies have highlighted the need for developing means of formally specifying and generating executable models of circuits that incorporate various types of brain data, including the heterogeneity and connectivity of different cells types and brain circuits, neurophysiology recordings as well as gene expression data. In principle, a whole brain simulation can be instantiated by modeling all the neurons and synapses of the connectome/synaptome with simple dynamics such as integrate-and-fire neurons and α-synapses, with parameters tuned according to certain criteria (Huang et al., 2019). Such an effort, however, may fall short of revealing the fundamental computational units required for understanding the functional logic of the brain, as the details of the units of computation are likely buried in the uniform treatment of the vast number of neurons and their connection patterns.

It is, therefore, imperative to develop a formal reasoning framework of the functional logic of brain circuits that goes beyond simple instantiations of flows on graphs generated from the connectome. A framework is needed for building a functional brain from components whose functional logic can be readily envisioned, and for exploring the computational principles underlying these components given the available data.

Recently released connectome, synaptome and transcriptome datasets of the *Drosophila* brain and ventral nerve cord (VNC) present a refreshing view of the study of neural computation (Zheng et al., 2018; Scheffer et al., 2020; Li et al., 2021). These datasets present challenges and opportunities for hypothesizing and uncovering the fundamental computational units and their interactions.

### 1.2. Modeling the Functional Logic of Fruit Fly Brain Circuits With Cell Types and Feedback Loops

The fruit fly brain can be subdivided into some 40 neuropils. The concept of the local processing unit (LPU) was introduced in the early works of the fly connectome to represent functional subdivisions of the fruit fly brain circuit architecture (Chiang et al., 2011). LPUs are characterized by unique populations of local neurons whose processes are restricted to specific neuropils.

It was not until the release of follow up electron microscopy (EM) connectome datasets that the minute details of the connectivity of these local neurons were revealed (Ohyama et al., 2015; Takemura et al., 2015; Zheng et al., 2018; Scheffer et al., 2020). Oftentimes, local neurons within each neuropil form intricate feedback circuits with a massive number of feedback loops.

**Figure 1:**
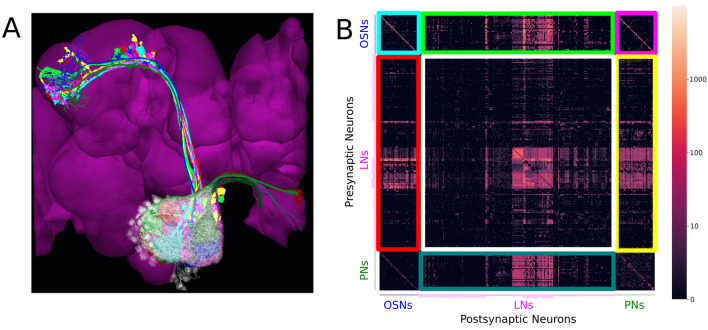
Massive number of feedback loops in the Antennal Lobe. **(A)** Antennal Lobe circuit involving OSNs (darker colors), PNs (brighter colors) and LNs (transparent white). Select OSNs, PNs and LNs are shown. **(B)** The adjacency matrix of the connectivity graph of the neurons in the AL, with all OSNs expressing the same OR merged into a single neuron group node, and all PNs in the same glomerulus merged into a single neuron group node. Matrix elements indicate the number of synapses from a presynaptic neuron (or neuron group) to a postsynaptic neuron (or neuron group). Magenta block on top right: submatrix of the feedforward connectivity from OSNs to PNs in each glomerulus. Green block on the top: submatrix of the feedforward connectivity from OSNs to LNs. Blue block on the bottom: submatrix of the connectivity from PNs to LNs. Red block on the left: submatrix of the feedback connectivity from LNs to OSNs. Yellow block on the right: submatrix of the feedback connectivity from LNs to PNs. White block in the middle: submatrix of the connectivity between LNs.

For example, the antennal lobe of the early olfactory system, consists of the axons of olfactory sensory neurons (OSNs) as inputs (depicted in Figure 1A in darker colors), the antennal lobe projection neurons (PNs) as outputs (in Figure 1A in brighter colors), and a large collection of local neurons (in Figure 1A in transparent white). The adjacency matrix of the connectivity graph of the AL circuit is shown in Figure 1B.

The axons of the OSNs expressing the same olfactory receptor (OR) project into the same glomerulus where they provide inputs to uniglomerular PNs (uPNs) whose dendrites only extend within the same glomerulus. Such connections form the feedforward signaling path in the antennal lobe (see the magenta-colored block in Figure 1B).

While not all neuropils share such glomerular structure, three features in the AL connectivity patterns can be found in many other neuropils.

First, OSNs expressing the same OR exhibit strong axon-axonal connections but not with OSNs expressing other ORs (see the cyan-colored block corresponding to the OSN-to-OSN connectivity on the top left of Figure 1B). Similar axonal connections can be observed between Kenyon Cells (KCs) of the mushroom body (MB) (Zheng et al., 2018), between Lobular Columnar (LC) neurons in the optic glomeruli (OG) (Scheffer et al., 2020), and between the ring neurons of the ellipsoid body (EB) (Hulse et al., 2021).

Second, local neurons in the AL can be grouped into a large number of cell types. The diversity of the LN cell types and the complexity of their arborization suggest the key role that the LNs play in shaping the functional logic of the AL. Determining the role each of these cell types plays is essential in modeling the functional logic of the AL circuit.

Third, local neurons receive inputs from OSNs and PNs (see green and blue blocks, corresponding to OSN-to-LN and PN-to-LN connectivity, respectively, in Figure 1B). They also provide feedback to OSNs and PNs (see red and yellow blocks, respectively, in Figure 1B). In addition, LNs also synapse onto other LNs (white block in Figure 1B). Given the simplicity of the feedforward signaling path and the complex nature of feedback driven by LN connectivity, the massive number of feedback loops must underlie the functional logic of the AL circuit.

A massive number of feedback loops can be ubiquitously found across other brain regions, for example in the medulla (Takemura et al., 2015), lateral horn, mushroom body (Scheffer et al., 2020), central complex (Hulse et al., 2021), etc. The feedback loops considered here map the states at the output of a circuit into inputs. Since the AL has a connectivity structure that in many ways is representative, for simplicity and clarity in the rest of this work we will be mostly focused on characterizing the AL circuit.

Finally, note that in mammals, particularly in the visual system, feedback pathways have long been considered to be a key component of the architecture of brain circuits (Lamme et al., 1998). However, due to the lack of detailed brain circuit connectivity in these higher organisms there remains insufficient insight into the functional role played by the feedback circuits. The connectome/synaptome of the fruit fly opens new avenues for discovering the full complexity and computational principles underlying feedback circuits.

### 1.3. A Programmable Ontology Encompassing the Functional Logic of the Fruit Fly Brain Circuits

Traditionally, ontologies formally define the classification of the anatomical structure of the *Drosophila* nervous system and the ownership relationships among anatomical entities (Costa et al., 2013; Lazar et al., 2021). However, existing ontologies lack computational primitives/motifs, such as feedback loops that can be more readily associated with the functional role of brain circuits.

Furthermore, characterizing the functional logic of sensory circuits calls for modeling the environment (“the input”) the fruit flies live in. The object structure of the space of natural sensory stimuli that the fruit flies constantly sample has not been discussed in the formal ontology of the fly brain anatomy. Although natural stimuli have been widely used in sensory neuroscience (Egelhaaf et al., 2002; Tootoonian et al., 2012; Jeanne et al., 2018), the modeling of the object structure of the environment (Lazar et al., 2022) has often been neglected in the neuroscience literature at large. The aforementioned object structure is, however, essential in defining, characterizing and evaluating the functional logic of brain circuits.

The goal of the work presented here is to accelerate the discovery of the functional logic of the fruit fly brain circuits. Programmability, in the age of connectomics/synaptomics, is key. Expanding the scope of the classical ontology to encompass the natural sensory stimuli and the functional logic of the *Drosophila* brain circuits bridges the gap between the two fields and greatly benefits both. To boot, a *programmable ontology* will provide a language not only for describing but also for executing the functional modules of, for example, the large number of cell types, the massive number of feedback loops observed in brain circuits, which contribute to making brain function more transparent. We believe that this programmable ontology will provide the foundation for exploring the functional logic of the brain.

**Figure 2:**
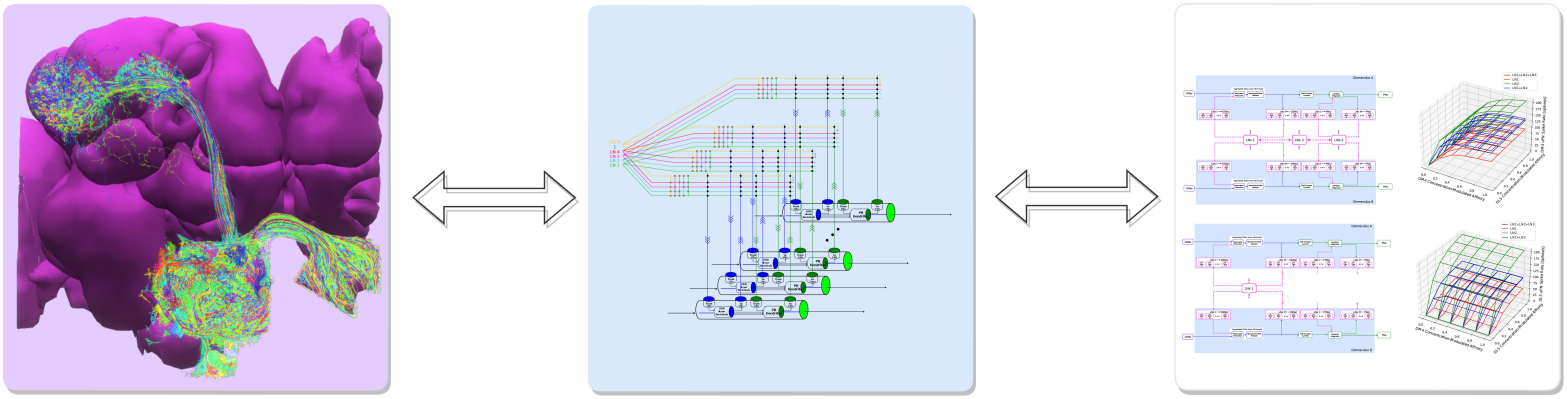
The workflow of discovery of the functional logic of the fruit fly brain. Left: 3D visualization and exploration of fly brain data. Middle: Creation of executable circuits. Right: Interactive exploration of the functional logic of executable circuits.

The proposed programmable ontology is tightly integrated with the workflow of the interactive FlyBrainLab (Lazar et al., 2021) computing platform, as elaborated in Figure 2.

The workflow in Figure 2 consists of 3 steps. First, 3D visualization (see Section 6) of fly brain morphology data is explored and candidate anatomical structures defining functional units and modules (Figure 2 left) identified. Second, the candidate biological circuits are mapped into executable circuits that provide an abstract representation of the circuit in machine language (Figure 2 middle). Third, the devised executable circuits are instantiated for the interactive exploration of their functional logic with a highly intuitive graphical interface for configuring, composing and executing neural circuit models (Figure 2 right, see Section 6).

The main rationale for the tight integration of the programmable ontology into the FlyBrainLab workflow of discovery is to fully anchor it onto biological data and the worldwide literature that describes it. FlyBrainLab fully supports the programmability of the ontology while easily supporting various computational schemes used for interrogating the functional logic of brain circuits.

## 2. Exploring the Morphology of Cell Types and Feedback Circuits

Recent releases of large-scale connectomic/synaptomic datasets have enabled experimental and computational neuroscientists to explore neural circuits in unprecedented detail. As Figure 2 suggests, understanding the functional logic of fruit fly brain circuits starts with the exploration of fly brain connectome/synaptome datasets. To efficiently explore these datasets requires, however, knowledge of both the biological nomenclature and programming tools. These skills are often limited to members of their respective communities. For example, neurobiologists who design new experiments often lack in-depth programming skills to efficiently explore these datasets. Even computational neuroscientists who perform neural circuit simulations may find the need to learn more recent database query languages that go beyond simple operations such as retrieving neurons by name. Computer scientists, working on the next generation of artificial neural networks that are informed by biological neural circuits, need to set aside a significant amount of time to learn the biological nomenclature.

To close the programming gap, we developed the natural language query interface NeuroNLP (Ukani et al., 2019; Lazar et al., 2021) to support highly sophisticated English queries of *Drosophila* brain datasets, including morphology and position of neurons (cell type map), connectivity between neurons (connectome) and distribution and type of synapses (synaptome). Moreover, NeuroNLP provided the first open neurophysiology data service for the fruit fly brain (activity map). However, the NeuroNLP rule-based query engine could only map pre-designed sentence structures into database queries, thereby limiting its usage. In particular, users unfamiliar with the nomenclature used in a dataset may have found it difficult to query for particular cell types.

In what follows, we introduce NeuroNLP++, a substantially upgraded NeuroNLP web application, that alleviates these limitations and helps users to explore fruit fly brain datasets with *free-form English queries*. In Section 2.1, we introduce the capabilities of the NeuroNLP++ application. In Section 2.2, we demonstrate the use of NeuroNLP++ to explore the morphology and graph structure of the cell types in the AL. In Section 2.3, we demonstrate how to use NeuroNLP++ to query feedback loops.

### 2.1. Key Capabilities of NeuroNLP++

Expanding upon the NeuroNLP query interface (Ukani et al., 2019; Lazar et al., 2021), NeuroNLP++ provides two additional key advances. First, NeuroNLP++ interprets and answers free-form English queries that are well beyond the natural language capabilities of NeuroNLP. Second, NeuroNLP++ not only visualizes neuron/synapses but also links them to the worldwide fruit fly brain literature.

This is achieved by associating descriptive terms of neurons of the fruit fly brain available in the open literature with connectomic datasets. For example, NeuroNLP++ integrates cell types or lineages from the *Drosophila* Anatomy Ontology (DAO) (Costa et al., 2013) and matches them against neurons in the Hemibrain connectome dataset (Scheffer et al., 2020). Given a query, NeuroNLP++ then employs state-of-the-art document retrieval techniques (Karpukhin et al., 2020) to find cell types whose description match the description in the query (see also Section 6).

**Figure 3:**
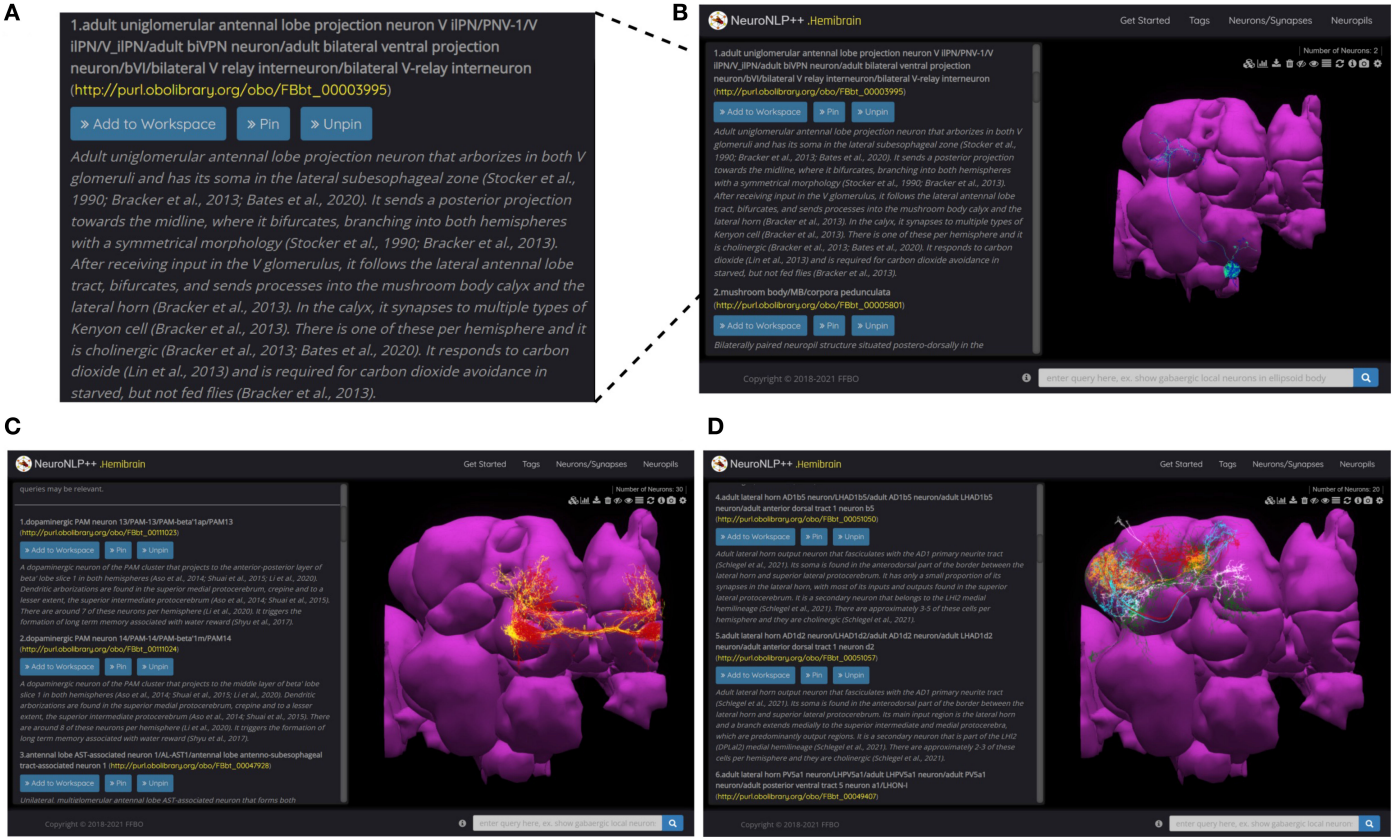
NeuroNLP++ user interface and query results. **(A)** Typical results displayed in the Info Panel of the NeuroNLP++ application in response to the query “which neurons respond to carbon dioxide?”. **(B)** The user interface of NeuroNLP++ with the Info Panel on the left and 3D visualization workspace on the right. **(C)** Results to the query “which neurons are associated with water reward?" with the first few relevant entries added to the workspace. (red) PAM13 neurons, (yellow) PAM14 neurons. **(D)** Results to the query “what cell types are there in lateral horn?” with the first few entries added to the workspace. (red) AD1b5 neurons, (green) LH centrifugal neurons, (cyan) AD1d2 neurons, (orange) PV5a1, (white) PD2a5 neurons.

These descriptions are reflected in the query results of NeuroNLP++ in response to a question also mentioned in the caption of Figures 3A,B. Here, we started by asking “what neurons respond to carbon dioxide?”. The query results, in the form of a list of the most relevant cell types, are displayed on the left of the NeuroNLP++ user interface, as shown in Figure 3B. Each entry lists the name of the cell type, a link to the DAO, as well as a description of the cell type. It also includes a UI button for adding to the workspace the neurons associated with the entry. The first query result, magnified in Figure 3A, includes names and synonyms of the V glomerulus projection neurons, as well as their ontological description along with specific entries to the relevant literature.

In Figures 3C,D, we present more examples of NeuroNLP++ queries. In Figure 3C we asked “which neurons are associated with water reward?,” and in Figure 3D “what cell types are there in lateral horn.” The query results revealed a variety of cell types. These may provide a starting point for exploring novel cell types associated with other neuropils and can guide additional rule-based queries. The two examples here can be found as live demos in the NeuroNLP++ application.

Compared with using NeuroNLP and other connectome-driven web services such as Neuprint (Clements et al., 2020), users benefit from employing NeuroNLP++ in several ways. First, with NeuroNLP++ neurons can be queried in ways that are not limited to the specific naming in a dataset. For example, a neuron may be named differently in different research papers, while a specific name is used in the Hemibrain dataset. Without knowing the specific name the neuron is called in the working dataset, the user may not be able to find with NeuroNLP the cell type by using a name mentioned in the literature. This knowledge of the nomenclature is not necessary when using NeuroNLP++. In addition, multiple matched results with descriptive answers alleviate the problem with naming ambiguity in English queries, when, for example, an abbreviation of the neuron name can refer to different cells in different brain regions.

Furthermore, NeuroNLP++ complements the specific sentence structure required by NeuroNLP. While the pre-designed sentence structure allows for querying neurons precisely by their properties, NeuroNLP++ allows questions to be more “open ended”. For example, querying cell types is not limited to their names, but a user can ask questions such as “what types of local neurons are in the antennal lobe?” and “what are the ring neurons?.”

Finally, NeuroNLP++ provides more context for the neurons searched in connectomic datasets by providing links to the worldwide literature associated with cell types. This will provide users, particularly those unfamiliar with the cell type literature, a convenient way of exploring prior knowledge.

### 2.2. Exploring the Morphology and Graph of Cell Types With NeuroNLP++

In addition to natural language querying capabilities, NeuroNLP++ provides an interactive Graph View application that displays the current neurons in the workspace at the neuronal or cell type level (see Section 6). While the morphology of neurons is often cluttered in the 3D visualization, Graph View helps sort out the connections between the neurons displayed.

**Figure 4:**
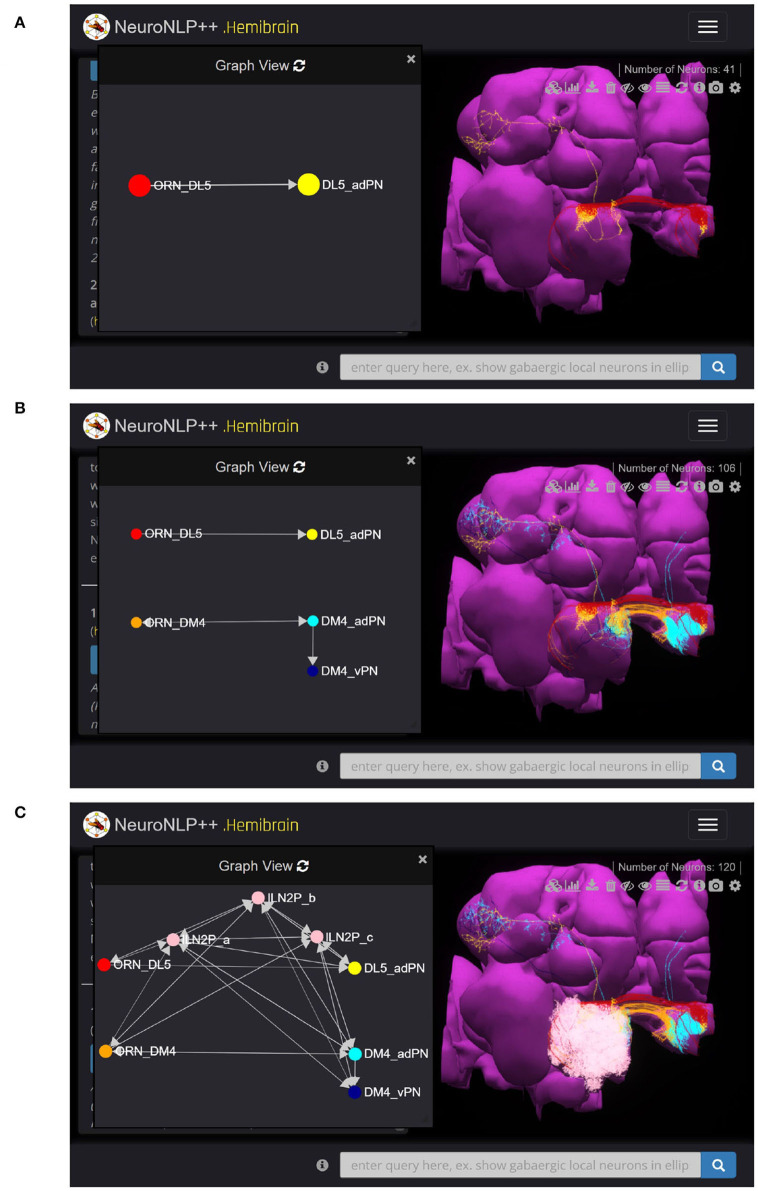
Free-form English queries of the AL with NeuroNLP++. **(A)** Result to the query “what are the cell types of the DL5 glomerulus?”, consisting of the OSNs with axons that arborize the DL5 glomerulus (red) and the PN with dendrites in the DL5 glomerulus (yellow). (left) Cell type connectivity graph of the visualized neurons. (right) Morphology of the retrieved neurons. **(B)** Result to the query “what are the cell types of the DM4 glomerulus?”, in addition to **(A)**, consisting of the OSNs (orange with axons that arborize the DM4 glomerulus and the adPNs (cyan) and vPNs (blue) with dendrites in the DM4 glomerulus. (left) Cell type level connectivity graph of the resulting neurons. (right) Morphology of the retrieved neurons. **(C)** Results to the query “what are the patchy local neurons?”, in addition to **(B)**. The resulting LNs are in pink. (left) Cell type connectivity graph of the visualized neurons. (right) Morphology of the retrieved neurons.

Here we use NeuroNLP++ to explore the morphology and Graph View to visualize the circuit diagram of several glomeruli in the AL. We started by asking “what are the cell types of the DL5 glomerulus”. The cell type Graph View of the neurons in the DL5 glomerulus is depicted on the left in Figure 4A. Here, the red and the yellow nodes represent the OSNs and PNs, respectively, and the arrow from the red to the yellow node indicates that the OSNs provide inputs to the PNs. The colors in Graph View match those in the 3D morphology visualization. Graph View is also interactive, allowing users to highlight the corresponding neurons in the 3D visualization.

We then asked “what are the cell types in the DM4 glomerulus”. The resulting neurons are added to the workspace and their cell type graph is depicted in Figure 4B. These include the OSNs (orange) that project into the DM4 glomerulus and two types of PNs, namely adPNs (cyan) that project to both the MB and LH, and vPNs (blue) that only project to the LH. The graph also confirms that the two glomeruli run in parallel.

Finally, we asked “what are the patchy local neurons?”. Patchy local neurons, typically arborize in a large number of glomeruli of the AL. They were first discovered in a light microscopy study of local neurons; information about their connectivity with neurons inside the glomeruli is lacking (Chou et al., 2010). The neurons obtained in response to our query are shown in white in Figure 4C, together with the cell type graph of the entire circuit. The connectivity graph suggests the presence of strong feedback components within and between the two otherwise disjoint glomerular circuits.

To explore the diversity of LNs in the AL, we needed to classify cell types based on their morphology. We launched the query “what are the types of local neurons in the antennal lobe?.” NeuroNLP++ provides a complete list of the currently known LN types in the AL. In Supplementary Figure S1 in Supplementary Material, we list all these LN types, the number of neurons of each type, and an example morphology of neuron type. The morphology of the neurons is colored by their glomerular arborization. The graph structure of a typical LN of each cell type is summarized in the matrix depicted on the right.

### 2.3. Exploring the Morphology of Feedback Circuits With NeuroNLP++

As discussed above, feedback loops are major targets of the study of the functional logic of the fruit fly brain. Consequently, in addition to querying cell types, we also built into NeuroNLP++ capabilities to query for neurons that belong to specific feedback loops.

Different feedback loops can be described as entities in the DAO. This enables NeuroNLP++ to search for feedback loops with English queries. We identified different types of feedback loops for each glomerulus (see Section 6) and further identified a number of specific feedback loops consisting of local neurons. For example, a circuit consisting of LNs that receive inputs from, and provide feedback to, OSNs but has no interaction with PNs, is named here an OSN-LN-OSN feedback loop. Similarly, a circuit consisting of LNs that receive inputs from, and provide feedback to, PNs but has no interaction with OSNs is named a PN-LN-PN feedback loop. A circuit consisting of LNs that receive inputs from and provide feedback to both OSNs and PNs is named an OSN/PN-LN-OSN/PN feedback loop.

**Figure 5:**
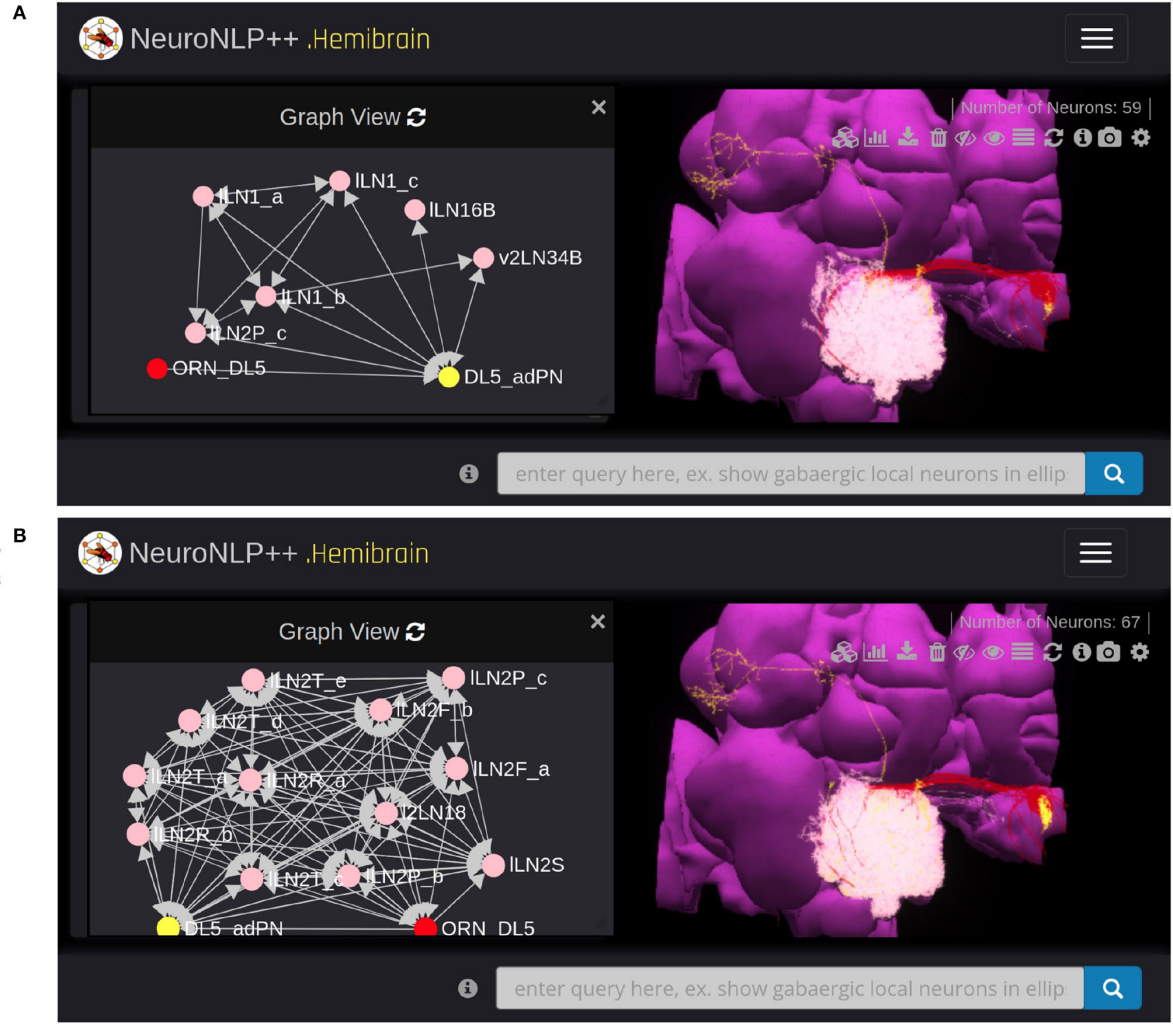
Exploring feedback loops with NeuroNLP++. The result to the query “show available feedback loops” when starting from Figure 4A with OSNs with axons that arborize the DL5 glomerulus and PNs with dendrites in DL5 glomerulus. **(A)** PN-LN-PN feedback loop (receives inputs from and only feeds back to PNs). (red) OSNs. (yellow) PNs. (pink) LNs. **(B)** OSN/PN-LN-OSN/PN feedback loop (LNs receive inputs from both OSNs and PNs and provide, respectively, feedback to them). (red) OSNs. (yellow) PNs. (pink) LNs.

To query for feedback loops associated with OSNs and PNs in the DL5 glomerulus, i.e., starting from Figure 4A, we requested: “show available feedback loops.” Figure 5 depicts two different types of feedback loops in response to this query.

In Figure 5A, we added the 19 LNs (pink) that form the PN-LN-PN feedback loop. From Graph View, we confirm that these feedback loops are only associated with the DL5 PNs (yellow) but not OSNs (red) node. The arrows into the adPN node indicate the feedback pathway from LNs into the adPN. In Figure 5B, we added the 26 LNs that form feedback loops with both OSNs and PNs. From Graph View we note that these LNs (pink) form feedback loops with both OSNs (red) and PNs (yellow) nodes.

Concluding, by establishing the NeuroNLP++ natural language query interface for exploring the morphology of fruit fly brain circuits, we effectively created an ontology of the fruit fly brain consisting of the existing anatomical ontology, the connectome/synaptome datasets and the published worldwide literature. Moreover, we provided visualization tools for extracting what are thought to be functionally significant circuits. NeuroNLP++ represents a step toward a more intuitive and natural way of extracting information from large connectome/synaptome datasets that are relevant for the in-depth study of the functional logic of brain circuits. In addition, the capability to anchor the queried connectome/synaptome data onto the published worldwide literature provides much needed awareness of the prior existing knowledge regarding these circuits.

## 3. Creating a Programmable Ontology of the Fruit Fly Brain

Our goal in this section is to demonstrate how the framework of the ontology outlined in the previous section can be further enriched and extended to encompass the key stimulus and processing elements needed for exploring the functional logic of the fruit fly brain circuits. Overall, the resulting ontology will be programmable from the ground up. Programmability calls for i) employing spaces of stimuli whose basic objects can be computationally modeled and identified, and ii) constructing brain circuit models using simple executable building blocks that are composable based on rules built upon and informed by biological entities such as cell types and feedback loops.

The significance of modeling the space of stimuli for characterizing the I/O of functional circuits arises throughout the early sensory systems, e.g., in early olfaction (Jeanne et al., 2018), vision (Egelhaaf et al., 2002), audition (Tootoonian et al., 2012), mechanosensation (Tuthill and Wilson, 2016), etc. The odorant space and the visual field are examples that come to mind. See, for example, Lazar et al. (2015) and Lazar and Yeh (2020).

Given the current connetomic datasets, we shall describe here how some of the better characterized neuropils can be modeled and constructed through a process of composability. Due to space limitations, we will only present in what follows a methodology of a receptor-centric modeling of the space of odorant stimuli, as well as a methodology for devising the olfactory processing in the antenna and the antennal lobe of the fruit fly brain. How to apply the same general methodology to other neuropils of the fruit fly brain entails a set of challenges that will be addressed elsewhere.

### 3.1. Receptor-Centric Modeling the Space of Odorant Stimuli

To fully characterize the functional logic of a sensory circuit calls for modeling the environment the studied organism lives in, a rather difficult undertaking. To model the environment, we first have to define the *space of odorant stimuli*. The space of odorant stimuli has never been discussed in the context of a formal ontology of the fruit fly brain anatomy. It is often neglected in the neuroscience literature, but essential in defining, characterizing and evaluating the functional logic of brain circuits involved in odor processing.

The Chemical Abstracts Service (CAS) registry has currently 156 million organic and inorganic substances registered (Morgan, 1965). Distinguishing between odorants in the CAS registry seems to be a problem of enormous complexity (Tran et al., 2019). How does the fly approach this problem? As a first step in the encoding process, the odorant receptors bind to the odorants present in the environment and that are of interest to the fly. The adult fruit fly has some 51 receptors whose binding and dissociation rates to/from odorant molecules characterize their identity. In addition to odorant identity, the odorant concentration amplitude is another key feature of the odorant space.

**Figure 6:**
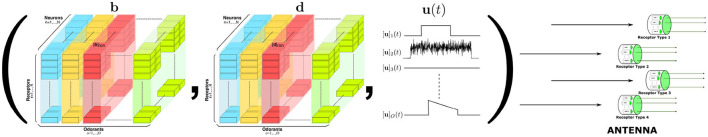
Elements of the odorant space are defined by the odorant-receptor binding rate, dissociation rate and concentration amplitude tensor trio [*b, d, u*(*t*)]. For a given neuron *n* = 1, 2, ..., *N*, the binding rate and dissociation rate values are, respectively, denoted by [*b*]_*ron*_ and [*d*]_*ron*_, for all *r* = 1, 2, ..., *R*, and *o* = 1, 2, ..., *O*. Single and/or odorant mixtures interact with the receptors expressed by the Olfactory Sensory Neurons in the Antenna (right).

The odorant space considered here consists of pure and odorant mixtures. Pure odorants are mostly used in laboratory settings for studying the capabilities and the function of the early olfactory circuits. Odorant mixtures widely arise in the living environment. Following (Lazar and Yeh, 2020), the identity of an odorant can be modeled by a 3D tensor pair (**b**, **d**). The 3D tensor **b** with entries [**b**]_*ron*_ is called the odorant-receptor binding rate and models the association rate between an odorant *o* and a receptor of type *r* expressed by neuron *n* (see also Figure 6). The 3D tensor **d** with entries [**d**]_*ron*_ denotes the *odorant-receptor dissociation rate* and models the detachment rate between an odorant *o* and a receptor of type *r* expressed by neuron *n* (see also Figure 6). We denote the odorant concentration waveforms as the vector **u**(*t*), where [**u**]_*o*_(*t*) denotes the concentration amplitude of odorant *o, o* = 1, 2, ⋯ , *O*. The odorant concentration can be any arbitrary continuous waveform (see also Figure 6). For a pure odorant 𝒪, [**u**]_*o*_(*t*) = 0, *o* ≠ 𝒪. A set of odorant waveforms modeled by the tensor trio (**b**, **d**, **u**(*t*)) is graphically depicted in Figure 6. Often, for simplicity, the binding rate [**b**]_*ron*_ and the dissociation rate [**d**]_*ron*_, for a given odorant *o* and a given receptor-type *r*, are assumed to take the same value for all neurons *n* = 1, 2, ..., *N*, expressing the same receptor-type *r*.

Note that the elements of the odorant space are not defined by the (largely intractable) detailed/precise chemical structure of the odorants. Rather, they are described by the rate of activation/deactivation between odorants and olfactory receptors. The tensor trio determines what types of sensors (olfactory receptors) will be activated by a certain odorant, and the level of activation will be jointly governed by the identity and the concentration waveform amplitude of the odorant. More precisely, for a single odorant, the overall activation of the sensors is determined by the value of the odorant-receptor binding rate modulated by the odorant concentration profile (Lazar and Yeh, 2020).

### 3.2. Building the Antenna Circuit With OSN Cell Types

The antenna circuit of the early olfactory system of the fruit fly consists of approximately 2,500 parallel Olfactory Sensory Neurons (OSNs) that are randomly distributed across the surface of the maxillary palps and antennae. In what follows, we will refer to the set of all OSNs on one side of the fruit fly brain as an antenna/maxillary palp (ANT) local processing unit (LPU).

The OSNs, depicted in Figure 6 (right) in groups based on the olfactory receptors (ORs) that they express, form parallel circuits. For simplicity, we assumed that the number of OSNs expressing the same receptor-type is *N*. OSNs in the same group are said to be of the same cell type.

For each OSN, the odorants are first transduced by an olfactory transduction process (OTP) that depends on the receptor-type (Lazar and Yeh, 2020). Each of the generated transduction currents drive biophysical spike generators (BSGs) that produce spikes at the outputs of the antennae (see Section 6). Note that unlike the OTP whose I/O characterization depends on the receptor-type, the BSGs of OSNs expressing different receptor-types are assumed to be the same.

### 3.3. Composing the Antennal Lobe Circuits With Cell Types and Feedback Loops

The overall goal of this section is to develop a methodology for modeling and constructing circuits of arbitrary complexity of the Antennal Lobe. The methodology demonstrated here is generalizable to the other neuropils in the early olfactory system of the fruit fly brain, including the mushroom body and the lateral horn; due to space limitations, the application of this methodology to the other neuropils of the early olfactory system will be presented elsewhere.

#### 3.3.1. Modeling Individual Glomeruli of the Antennal Lobe Circuit

As sketched in Figure 4, the AL exhibits a glomerular structure. Each glomerulus is primarily driven by the feedforward connections between the OSNs expressing the same OR and the corresponding PNs. As already mentioned, in addition to OSNs and PNs, LNs are the third cell type in each glomerulus. Although the modeling of glomeruli presented in this section is rather general, the concrete examples given below revolve around the DM4 and DL5 glomeruli.

**Figure 7:**
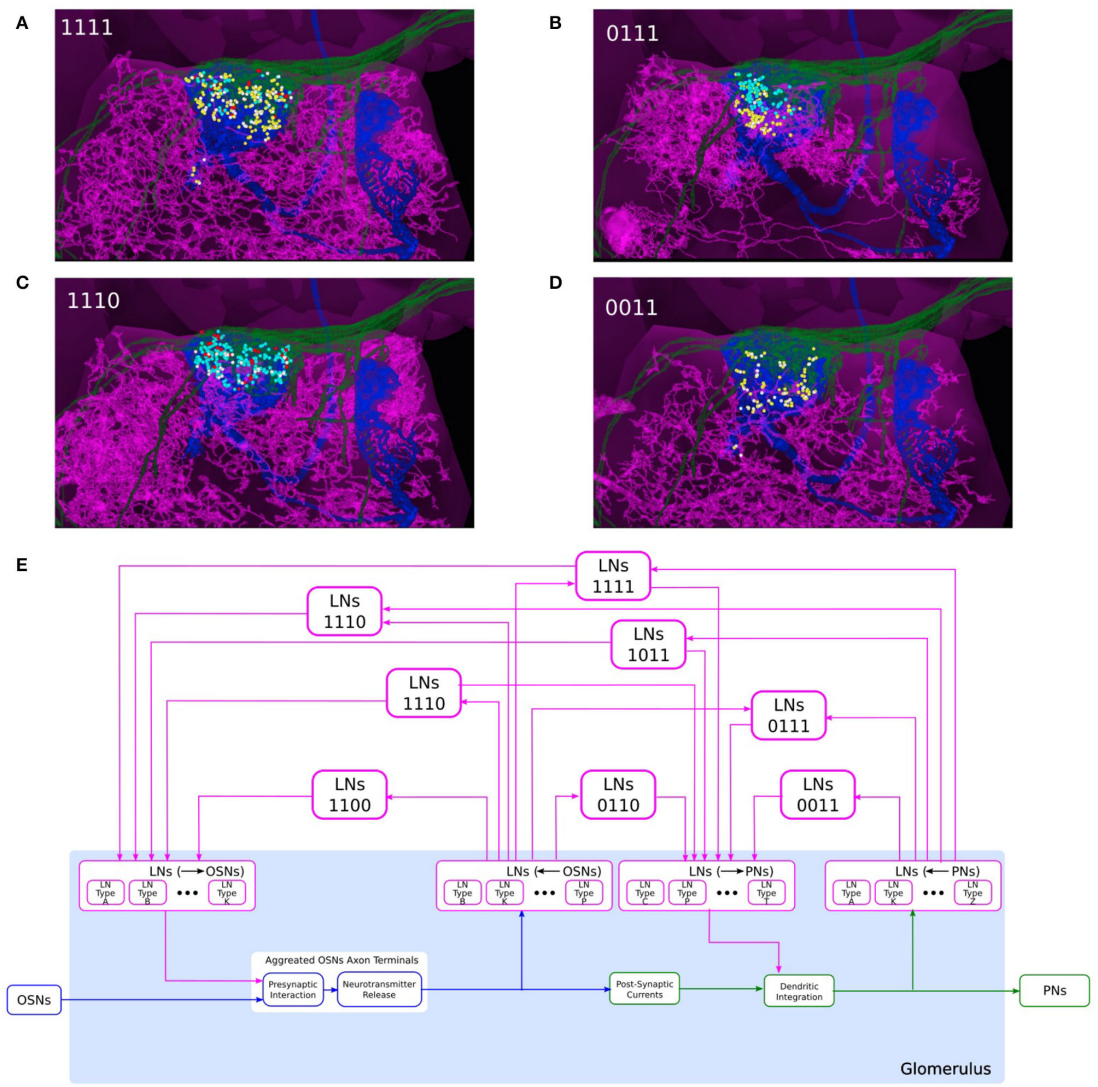
Composability of the port connectivity of LNs within a glomerulus. **(A–D)** Examples of LNs with different port connectivity patterns in the DL5 glomerulus. **(A)** 1111, **(B)** 0111, **(C)** 1110, and **(D)** 0011 (see also (E)). (magenta) LN, (green) the OSNs that project to DL5 glomerulus, (blue) the PN that innervates the DL5 glomerulus, (cyan dots) locations of OSN-to-LN synapses, (red dots) locations of LN-to-OSN synapses, (yellow dots) locations of PN-to-LN synapses, (white dots) locations of LN-to-PN synapses. **(E)** Schematic diagram of a single glomerulus circuit. (bottom) The blocks at the very bottom of the glomerulus represent the feedforward circuits. (top) LNs with different port connectivity patterns. Port connectivity patterns that have less than 50 occurrences in the Hemibrain dataset are omitted (see also Supplementary Table S1 in Supplementary Material).

To model a glomerulus, we closely followed the connectomic data provided by the Hemibrain dataset (Scheffer et al., 2020). In what follows the emphasis will be on showing how to extract/model the connectivity among cell types. We created a circuit diagram as depicted in Figure 7E. Here we abstracted the group of OSNs with axons that arborize the glomerulus as a single OSN (cell type). Similarly, we abstracted the group of PNs with dendrites in the same glomerulus as a single PN (cell type) (trivially obtained for DL5 since this glomerulus features a single PN). We only considered here the PNs that send their axons to both the MB and LH, and, thereby, primarily omit the vPNs with axons that only arborize the LH but not the MB. As shown in Figure 4C for the DM4 glomerulus, the omitted PNs typically receive inputs from other PNs rather than OSNs.

Since the OSN axon terminals and PN dendrites arborize the respective glomerulus, their synaptic connections with LNs must also occur within the same glomerulus. This is detailed in the examples in Figures 7A–D for the DL5 glomerulus. OSNs with axons arborizing in the DL5 glomerulus are shown in green, the PN with dendrites arborizing the same glomerulus is shown in blue, and 4 different LNs are shown in magenta, respectively. The locations of synapses between the OSNs and the LNs, and between the PN and the LNs are respectively shown in colored circles. The LN in Figure 7A receives inputs from the OSNs (cyan dots), provides inputs to the OSNs (red dots), receives inputs from the PN (yellow dots) and provides inputs to the PN (white dots). The LN in Figure 7B does not provide inputs to the OSNs; the LN in Figure 7C lacks PN inputs; the LN in Figure 7D does not synapse onto and receives no inputs from the OSNs shown.

Therefore, in the circuit diagram of the glomerulus in Figure 7E, we included 4 types of connections between OSNs and LNs and between PNs and LNs, including i) LNs presynaptically linked with OSN axon terminals, ii) LNs receiving inputs from OSN axon terminals, iii) LNs providing inputs to PNs, and iv) LNs receiving input from PN dendrites. Within the glomerulus, however, we do not specify the exact LNs that carry out these interactions. Rather, we define 4 ports (see the magenta blocks in Figure 7E): i) LNs (→ OSNs), ii) LNs (←OSNs), iii) LNs (→ PNs), and iv) LNs (←PNs), corresponding to, respectively, the 4 types of connections mentioned above. The connections from/to the specific LNs will be defined through these ports. All the LNs that connect to each port carry out the specific port connectivity pattern within the glomerulus.

The innervation of an LN within a glomerulus can then be graphically composed using the 4 ports. There are 15 different patterns of port connectivity within a glomerulus, that we call LN port connectivity patterns. We use a 4 digit binary code to represent this connectivity, according to the left-right order of the ports in Figure 7E. For example, if an LN receives inputs from OSNs and provides feedback to the same OSNs, but has no input from or output to PNs, then we call this port connectivity pattern “1100.” The number of occurrences of each port connectivity pattern according to the Hemibrain dataset can be found in Supplementary Table S1 in Supplementary Material. Note that a single LN can engage into different port connectivity patterns with different glomeruli.

If the LN innervation in a glomerulus follows the port connectivity pattern 11xx, xx11 or 1xx1, then the said LN is considered to form a feedback loop within the glomerulus. Eight out of 15 port connectivity patterns are associated with feedback, and they are shown in Figure 7E. The rest of the port connectivity patterns are only involved in feedback across glomeruli (see below).

#### 3.3.2. Modeling and Constructing Interconnected Glomeruli of the Antennal Lobe Circuit

For individual glomeruli, we have introduced 4 ports whose composability in the form of port connectivity patterns allow us to construct local feedback circuits. Here, we introduce composition rules of interconnected glomeruli that are also based on port connectivity patterns. The composability of port connectivity patterns enable scaling to multiple glomeruli. Furthermore, their programmability strengthens the reach of exploration of the functional logic of models of brain circuits.

**Figure 8:**
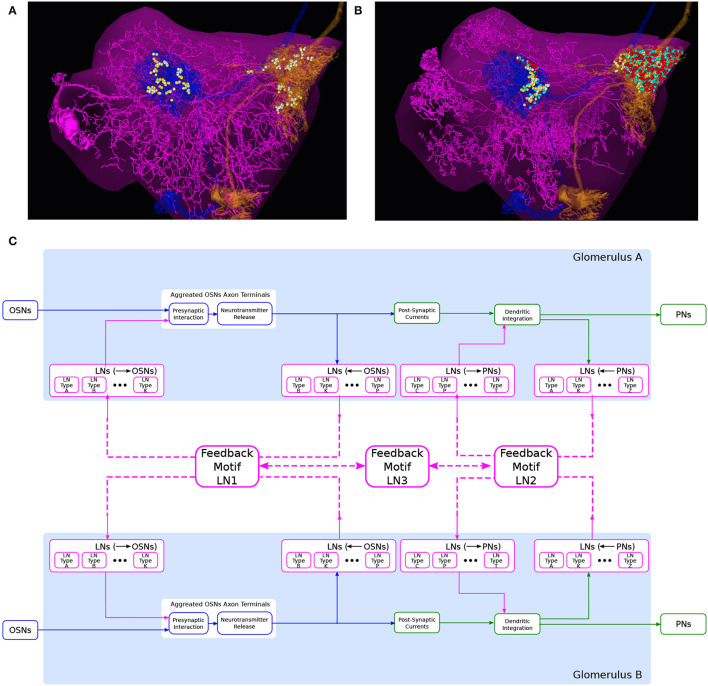
An abstraction of glomerular feedback circuits with 3 feedback motifs. **(A)** An LN that is presynaptic and postsynaptic to the PNs, but not to OSNs, in both DL5 and DM4 glomeruli. (magenta) the LN, (blue) a PN with dendrites arborizing the DL5 glomerulus, (orange) a PN with dendrites arborizing DM4 glomerulus, (yellow dots) locations of PN-to-LN synapses, (white dots) locations of LN-to-PN synapses. **(B)** An LN that is presynaptic and postsynaptic to the OSNs and PNs in both DL5 and DM4 glomeruli. Color is the same as in **(A)** and additionally, (cyan dots) locations of OSN-to-LN synapses, (red dots) locations of LN-to-OSN synapses. **(C)** A circuit diagram of two glomeruli with 3 feedback motifs. Feedback motif LN1 corresponds to the port connectivity patterns 1100 of each of the two glomeruli. Feedback motif LN2 corresponds to the port connectivity patterns 0011 of each of the two glomeruli, e.g., **(A)**. Feedback motif LN3 models the bidirectional connectivity between LN1 and LN2.

To compose the “wiring diagram” of glomeruli, we define feedback motifs using the port connectivity patterns that an LN links and that belong to distinct glomeruli. Here, we provide a number of example feedback motifs based on two interconnected glomeruli, as depicted in Figure 8C.

The first example feedback motif is based on LNs that link the port connectivity pattern 0011 of each of the 2 glomeruli. This is denoted LN2 in Figure 8C. An instance of such an LN is depicted in Figure 8A. The second example feedback motif is an LN that links the port connectivity pattern 1111 of each of the 2 glomeruli. An instance of such LN is depicted in Figure 8B (omitted in Figure 8C). Continuing to do so, we can create a collection of such feedback motifs based on combinations of port connectivity patterns. Note that the patterns are not necessarily the same on both glomeruli.

Composability also allows us to create feedback motifs that are not present in the connectome but that can still be of interest in studying the computational role of certain feedback loops. For example, the feedback motif presented as LN1 in Figure 8C has the port connectivity pattern 1100 of each of the 2 glomeruli.

Finally, we define a feedback motif LN3 that models the port connectivity between LNs. The LN3s do not receive or feedback to either OSNs or PNs. Rather, they connect only with the other feedback motifs.

## 4. Exploring the Functional Logic of Feedback Circuits in the Antennal Lobe

In this section, we present an approach for exploring the functional logic of feedback circuits of the fruit fly brain. This pertains to the third column of the workflow diagram of Figure 2. Specifically, we present here the interactive exploration of the AL following the previous sections where the morphology of the AL feedback circuits has been explored (Section 2) and single as well as pairwise interconnected glomeruli modeled and constructed (Section 3) (see also the second column of Figure 2).

We describe a programming library for instantiating examples of Antennal Lobe cell types and feedback circuit motifs abstracted from connectome data with customizable parameters of neurons and synapses. We demonstrate in Section 4.1 the use of this circuit library in exploring the I/O of a single glomerulus and in Section 4.2 for a pair of interconnected glomeruli. In Section 4.3, we then provide an example of scaling the methodology presented here for exploring the functional logic of the entire AL circuit.

### 4.1. Exploring the Functional Logic of Feedback Circuits of a Single Glomerulus in Isolation

For clarity in the presentation and simplicity in evaluation, in this section we build upon the composability of LNs within a glomerulus detailed in Section 3.3 and shown in Figure 7.

**Figure 9:**
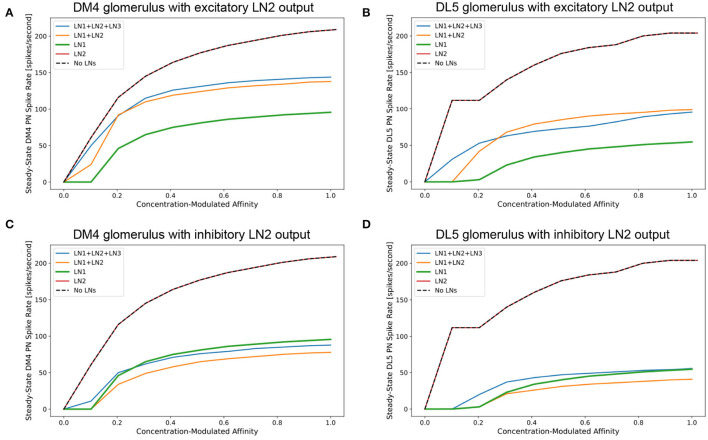
Characterization of PN responses of the single (isolated) DM4 and DL5 glomerular circuits. **(A)** DM4 PN steady-state firing rate across different constant odorant concentration levels. (dashed black) No LN is present. (green) Only LN1 feedback motif is present. (red) Only LN2 feedback motif is present. (orange) LN1 and LN2 feedback motifs are present. (blue) LN1, LN2 and LN3 feedback motifs are present. **(B)** DL5 PN steady-state firing rate across different constant odorant concentration levels. LN2 feedback motifs in both **(A,B)** are assumed to be excitatory. **(C)** DM4 PN steady-state firing rate when LN2 feedback motifs are assumed to be inhibitory. **(D)** DL5 PN steady-state firing rate when LN2 feedback motifs are assumed to be inhibitory.

In Figure 9, we evaluate the I/O behavior of the DM4 and DL5 glomeruli separately and in isolation for different compositions of feedback motifs. We outline how the presence of different feedback motifs can jointly or individually alter the PN output of the glomeruli. In our experimental setup, the number of OSNs and PNs, as well as the number of synapses between a given OSN and PN are configured using the data from the Hemibrain connectome dataset.

In Figures 9A,C, we evaluate and compare the DM4 glomerulus response due to different compositions of feedback motifs. We added to the single DM4 glomerular circuit composed of the OSNs, PN and three feedback motifs: LN1, LN2, and LN3 (see also Section 6). Results shown in Figure 9A were obtained assuming that the output of the feedback motif LN2 is excitatory, while for those in Figure 9C the output of the feedback motif LN2 is inhibitory.

For constant odorant concentration waveforms, the steady-state firing rates of the corresponding PNs are displayed in Figure 9A (see also Section 6). For the range of modulated affinity values, if the glomerulus is configured without any feedback loops, then the PN is driven immediately to saturation (Figure 9A dashed black curve). The addition of the feedback motif LN1, that presynaptically inhibits OSNs, results in a sigmoidal PN spiking rate for the tested range of odorant concentration values (Figure 9A green curve). We also note that the addition of the feedback motif LN2 alone, either with excitatory or inhibitory output, does not directly contribute to regulating the PN response, and results in a saturation level similar to the circuit without feedback (Figures 9A,C red curves).

We evaluated next the effect of the composition of feedback motifs. The orange curves in Figures 9A,C depict the responses of DM4 PNs when feedback motif LN2 with, respectively, excitatory and inhibitory outputs is added to the circuit with the feedback motif LN1 already present. Finally, we added feedback motif LN3 to the previous setup and stimulated it externally with a 20 nA current source. Injecting an external current enabled us to explore how activation of this feedback motif affects the responses of the DM4 PNs; this results in regular spiking in LN3 and suppression of both feedback motifs LN1 and LN2. However, the suppression of LN2 causes a larger effect and thus a net decrease in the PN spiking rate. These simple explorations demonstrate the effect different feedback loop compositions might have on the responses of the one-glomerulus circuit.

Evaluations with the same set of compositions were performed on the feedback circuit of the DL5 glomerulus (Figure 9B where output of LN2 is excitatory and Figure 9D where output of LN2 is inhibitory). Here, we observed similar contributions from different compositions of the feedback motifs as in the case of the DM4 glomerulus. Results for DM4 and DL5 if the output of LN2 is inhibitory are shown in Figures 9C,D, respectively. As expected, in this scenario, removing feedback motif LN2 causes a higher spike rate of the PN. An indirect suppression of LN2 through stimulation of LN3 similarly raises the spike rate of the PN.

The comparison of the PN outputs using different feedback motifs (Figures 9A–D) shows that i) the feedback motif LN1 is essential for the circuit to be stable under a large range of concentration amplitude values, ii) the addition of the feedback motif LN2 amplifies the steady-state PN spike rate response given the presence of the feedback motif LN1, and feedback motif LN3 controls the contribution of LN1 and LN2 to the I/O behavior of the circuit.

### 4.2. Exploring the Functional Logic of Feedback Circuits of a Pair of Interconnected Glomeruli

For clarity in the presentation and simplicity in evaluation, we shall use in this section the feedback circuit motif examples of the pair of interconnected glomeruli presented in Section 3.3 and detailed in Figure 8.

**Figure 10:**
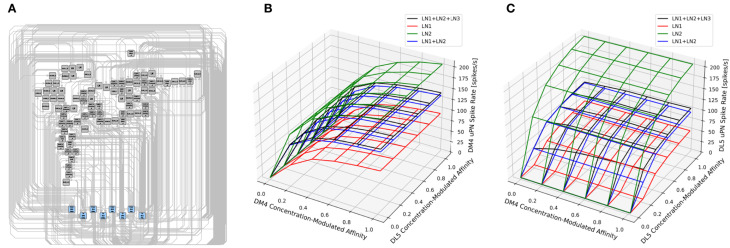
Characterization of DM4 PN and DL5 PN responses of a circuit consisting of interconnected DM4 and DL5 glomeruli. **(A)** A circuit diagram of the interconnected DM4 and DL5 glomeruli. Toward the bottom, the blue nodes represent ports to and from DM4/DL5 OSNs/PNs. Each gray node corresponds to a specific LN in the Hemibrain dataset. Only connectivity between the gray nodes and the blue nodes are shown in the diagram. **(B)** DM4 PN steady-state firing rate and **(C)** DL5 PN steady-state firing rate as glomeruli are subject to different concentration modulated affinity values. For both **(B,C)**: (red) Only LN1 feedback motifs are present. (green) Only LN2 feedback motifs are present. (blue) LN1 and LN2 feedback motifs are present. (black) LN1, LN2 and LN3 feedback motifs are present.

In Figure 10, we evaluated a circuit consisting of a pair of interconnected DM4 and DL5 glomeruli. A circuit diagram of the interconnected DM4 and DL5 glomeruli is shown in Figure 10A. The number of OSNs and PNs in these two glomeruli, and the number of synapses between these two types of neurons are configured according to the Hemibrain dataset. Following the motifs we explored in Figure 8, we then added five feedback circuit motifs: 1 LN1 each connecting to only DM4 and DL5, 1 LN2 each connecting to only DM4 and DL5, and 1 LN3 bidirectionally connected to LN1 and LN2 (see also Section 6).

In Figures 10B,C, we show the average spike rate of, respectively, the DM4 PN and DL5 PN as a function of the input value. Mirroring Figure 9, we consider compositions of different feedback circuit motifs. As in Figure 9, we find that the addition of LN1 alone produces the lowest spiking rate (Figures 10B,C red mesh). Similarly, the addition of LN2 alone produces the highest spiking rate due to lack of presynaptic inhibition from LN1 (Figures 10B,C green mesh). When we added feedback motif LN2 in addition to feedback motif LN1, the excitatory nature of the LN2 loop resulted in a spike rate increase by a small amount when compared with the DM4 glomerulus with only the feedback motif LN1 (Figures 10B,C blue mesh). Finally, by adding the feedback motif LN3 to the previous setup, the spike rate for the DM4 or DL5 glomerulus increases with the affinity of the receptors expressed by the OSNs projecting into the respective glomerulus. Note that, an increase in the odorant amplitude waveform of the OSNs projecting to one of the glomeruli also affects the spike rate of the other through the feedback motif LN3 (Figures 10B,C black mesh).

### 4.3. Circuit Library for Exploring the Functional Logic of the Massive Number of Feedback Loops in the Antennal Lobe

**Figure 11:**
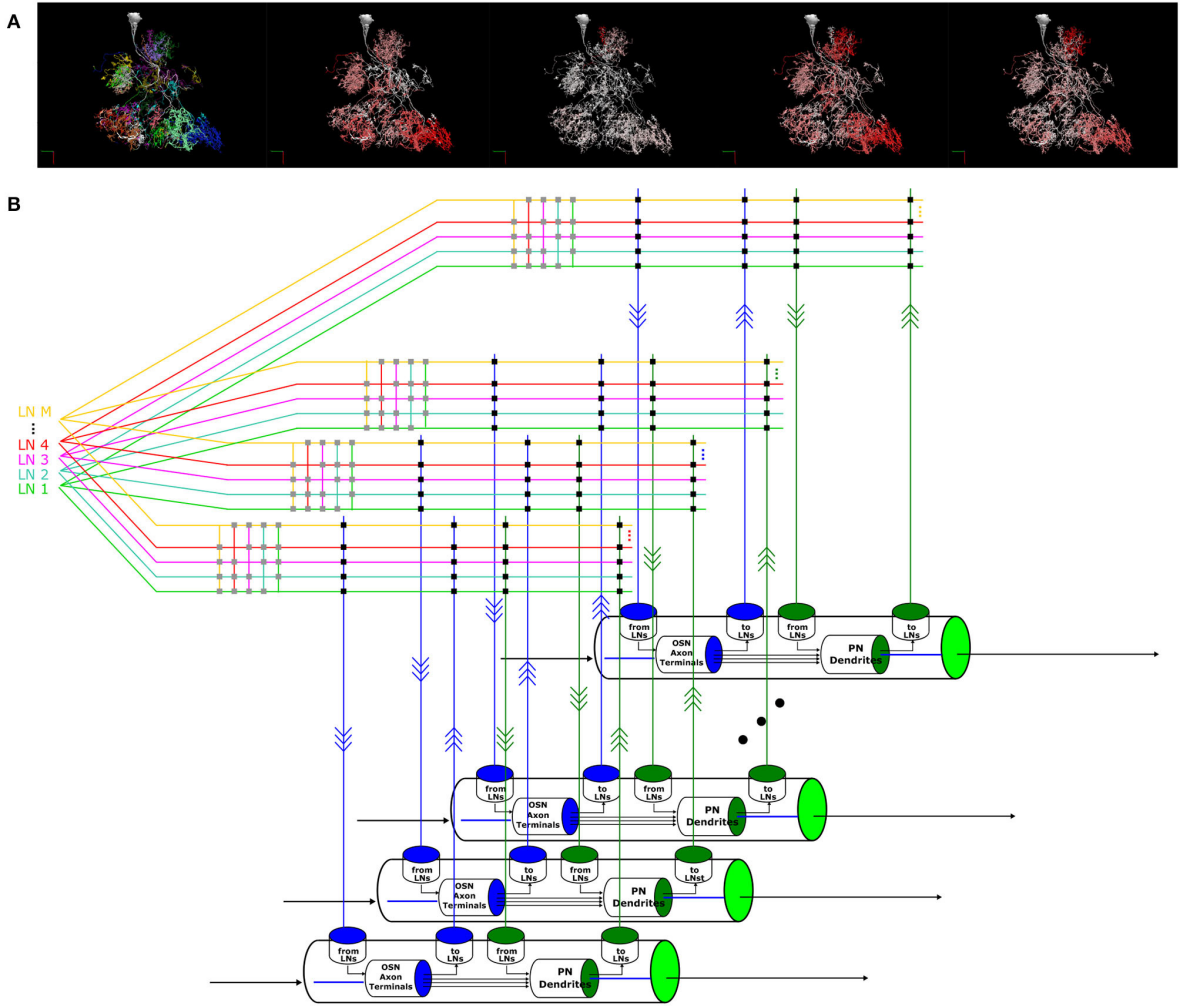
A circuit diagram modeling the entire AL circuit. **(A)** Morphology of an LN, its innervation of glomeruli and its connectivity to the glomeruli it innervates. The neurites in each glomerulus are colored differently, and from left to right: 1) Each color indicates the glomerulus the LN arborizes. 2) The arbors of the LN in each glomerulus are colored in red with saturation proportional to the number of synapses with the OSNs with axons arborizing in that glomerulus, i.e., redder indicates higher number of synapses. 3) The arbors of the LN in each glomerulus are colored in red with saturation proportional to the number of synapses onto the OSNs with axons arborizing in that glomerulus. 4) The arbors of the LN in each glomerulus are colored in red with saturation proportional to the number of synapses with the PNs in that glomerulus. 5) The arbors of the LN in each glomerulus is colored in red with saturation proportional to the number of synapses onto the uPNs in that glomerulus. **(B)** Schematic diagram of the glomeruli and the massive number of feedback circuits in the AL.

With models of OSN and PN cell types, LN feedback motifs, single and pairwise interconnected glomeruli, a methodology to address the composibility of feedback circuits has emerged that can be generalized to the entire AL. In particular, the morphological LN types described in Section 2.3 provide a blueprint for connecting multiple glomeruli via the LNs. Figure 11A depicts one such LN that innervates more than 20 glomeruli. In Supplementary Figure S1 in Supplementary Material, we list all these morphological LN types identified in the Hemibrain dataset, the number of neurons of each type, and an example neuron-type morphology. The morphology of the neurons is colored by their glomerular arborization.

To model the entire AL feedback circuit, we define glomeruli as parallel channels (Scott and Dahanukar, 2014) each exposing 4 ports that were depicted in Figures 7E, 8. The ports and the LNs form a crossbar as depicted in Figure 11B, where the crossbar “intersections” are marked with black dots. The connections of each LN with the ports of glomeruli are determined by the matrix listed on the right of Supplementary Figure S1. LNs also form a second crossbar associated with each glomerulus, depicted in Figure 11B, where the crossbar “intersections” are marked with gray dots. The exact connectivity pattern can be constructed through compositions.

To formally address composability of the circuit models of the programmable ontology, we introduce here a circuit library, called FeedbackCircuits, for exploring the functional logic of the massive number of feedback loops (motifs) in the fruit fly brain. While the library is generic and can applied to any local processing unit of the fly brain, we highlight here its capabilities in constructing and exploring the AL feedback circuit models described in Section 3.

First, the FeedbackCircuits Library provides tools for interactively visualizing and exploring the feedback loops in the AL circuit operational on the FlyBrainLab computing platform (Lazar et al., 2021) (see Section 6 for details).

Second, the FeedbackCircuits Library enables users to instantiate an executable circuit of the feedback circuit model in two ways. An executable circuit can be instantiated according to a connectome dataset. For example, any circuit explored via NeuroNLP++ can be loaded into an executable circuit directly. It can also be instantiated according to the abstraction of feedback motif examples provided in Section 3.3 (see also Section 6).

While the connectivity pattern of neurons can be extracted from connectome datasets, users can also define higher level objects, such as glomeruli of the AL (see also Section 6). Within a chosen object, the characteristics of the executable models, such as the dynamics of neurons of different cell types, are specified by the user. For example, users can specify all OSN to PN connections to execute commonly-used models of synaptic dynamics. Every instance of such synapses, residing in the connectome dataset, will be automatically assigned the specified dynamics. Similarly, all LN to OSN connections can be specified to act presynaptically on OSN axon terminals (Lazar et al., 2020).

Finally, different LN feedback motifs can be flexibly configured, stimulated or ablated in the FeedbackCircuit Library and their individual and combined effect on the AL outputs can be evaluated.

The FeedbackCircuits Library provides easy-to-customize loader and visualization functions to explore the I/O behavior of the antennal lobe circuit. This process can be repurposed for a wide variety of neuropils, including the mushroom body and the lateral horn of the early olfactory system.

## 5. Discussion

### 5.1. A Programmable Ontology Encompassing the Functional Logic of the Brain

Existing neuroscience-centered ontologies, including those of the fruit fly (Costa et al., 2013), the rat/mouse (Paxions and Watson, 2013; Swanson, 2018; Wang et al., 2020) and the human (Sunkin et al., 2012) brain, mainly focus on neuroanatomical structures, hierarchies and nomenclature. Description of entities or relations that have functional significance is rare and is kept at behavioral or cognitive level (Poldrack and Yarkoni, 2016).

While describing the structure of the brain is certainly a first step in the quest of understanding brain function, it is far from being sufficient. Thus, an ontology of the brain can not end with the description of anatomical data. Rather, the anatomical entities and relations have to be augmented with insights characterizing the functional logic of brain circuits.

In this paper, we presented a programmable ontology that expands the scope of the current ontology of *Drosophila* brain anatomy (Costa et al., 2013; Lazar et al., 2021) to encompass the functional logic of the fly brain. The programmable ontology provides a language not only for modeling circuit motifs but also for programmatically exploring their functional logic. To achieve this goal, we tightly integrated the programmable ontology with the workflow of the interactive FlyBrainLab computing platform (Lazar et al., 2021). In effect, the programmable ontology, embedded into the FlyBrainLab, has grown into a programming environment operating with access to a plethora of datasets, containing models of sensory space, the connectome/synaptome including cell types/feedback loops and neuronal/synaptic dynamics. The programmable ontology has the “built in” capability for evaluating the functional logic of brain circuits and for comparing their behavior with the biological counterparts.

To provide a language for defining functional circuit motifs anchored onto biological datasets and the worldwide literature, we developed the NeuroNLP++ web application that supports free-form English query searches of ontological entities and references to these in the published literature worldwide. NeuroNLP++ enables circuits to be composed using connectomic/synaptomic data in support of the evaluation of their function *in silico*. To bridge the gap between the existing *Drosophila* Anatomy Ontology dataset and the Hemibrain connectome morphology dataset, we associated with each ontological entity the corresponding neurons in the morphology dataset. The DrosoBOT Engine, in conjunction with the rule-based NLP engine, represents a first step toward providing a unified and integrated view of connectomic/synaptomic datasets and of the fruit fly brain literature worldwide.

In our programmable ontology the modeling of the space of sensory stimuli is explicitly included. We note that, e.g., the space of odorants has not been discussed in formal ontologies of the fly brain anatomy, although it plays a key role in defining, characterizing, and evaluating the functional logic of brain circuits. Here, the odorant space is modeled by a 3D tensor trio that describes the interaction between odorants and olfactory receptors, rather than by the (largely intractable) detailed/precise chemical structure of the odorants. Defining odorants and odorant mixtures as well as their interactions with olfactory receptors is an important step of this program.

By augmenting the ontology with the space of odorant objects and by providing an English query web pipeline for exploring structural features of the architecture of the early olfactory brain circuits, we are now in a position to evaluate the functional logic of these circuits in their full generality. The program of research presented here, due to space limitations, only sets the stage to modeling and exploratory computational evaluation of the early olfactory system. Clearly, an extension to the other sensory modalities is in order. In particular, the early vision system (Lazar et al., 2015) and the central complex (Givon et al., 2017; Lazar et al., 2021) are our next candidates.

### 5.2. Construction of Circuit Motifs With the FeedbackCircuits Library

Detailed connectomic datasets, such as the Hemibrain dataset, reveal a massive number of nested feedback loops among different cell types. Dissecting the role of these feedback circuits is key to the understanding the model of computation underlying the Local Processing Units (LPUs) of the fruit fly brain. The methodology underlying the FeedbackCircuits Library we advanced here has wide reaching implications for studying the massive feedback loops that dominate all regions/neuropils of the fruit fly brain.

The FeedbackCircuits Library brings together the available *Drosophila* connectomic, synaptomic and cell type data, with tools for 1) querying connectome datasets that automatically find and incorporate feedback pathways, 2) generating interactive circuit diagrams of the feedback circuits, 3) automatic derivation of executable models based on the composition of feedback motifs anchored on actual connectomic data, 4) arbitrary manipulation (and/or ablation) of feedback circuits featured by an executable interactive circuit diagram, and 5) systematic characterization and comparison of the effect of different feedback loops on the I/O behavior of arbitrary brain circuits.

We have demonstrated the capabilities of the FeedbackCircuits Library using circuits of the DM4 and DL5 glomeruli of the *Drosophila* antennal lobe constructed, based on the Hemibrain dataset, either in isolation or pairwise interconnected. We have also demonstrated a methodology for constructing, exploring and characterizing the contribution of individual feedback motifs as well as their compositions. The entire AL feedback circuit can be readily constructed with the approach we outlined.

The work presented here represents the beginning of an in-depth study of feedback motifs and their functional logic. By outlining the programmable ontology and demonstrating its workflow in exploring the functional logic of brain circuit from fly brain data, we advanced an accelerated path for the exploration and discovery of the functional logic of the fruit fly brain.

Studies of the functional logic of sensory processing neuropils such as the medulla and the mushroom body are currently limited to the feedforward pathways (Yang and Clandinin, 2018; Borst et al., 2020; Modi et al., 2020), although these circuits exhibit strong feedback components (Nern et al., 2015; Eschbach et al., 2020; Lazar et al., 2021). Our methodology paves the way for deeper investigations into the composition of such feedback circuits.

## 6. Materials and Methods

In this section, we present the details of the methodology for the exploration of the morphology of massive feedback circuits, modeling odor signal processing in the early olfactory system, and the interactive exploration of the antennal lobe as a network of glomeruli.

For creating the tools underlying the programmable ontology we used extensive capabilities to query datasets and build executable circuits, query the antennal lobe circuitry using these as well as customized tools, constructing and evaluating the feedback circuits with the FeedbackCircuits Library, and mapping glomeruli and their compositions into executable circuits.

### 6.1. Exploring the Morphology of Cell Types and Feedback Circuits

#### 6.1.1. The NeuroNLP++ Web Application

**Figure 12:**
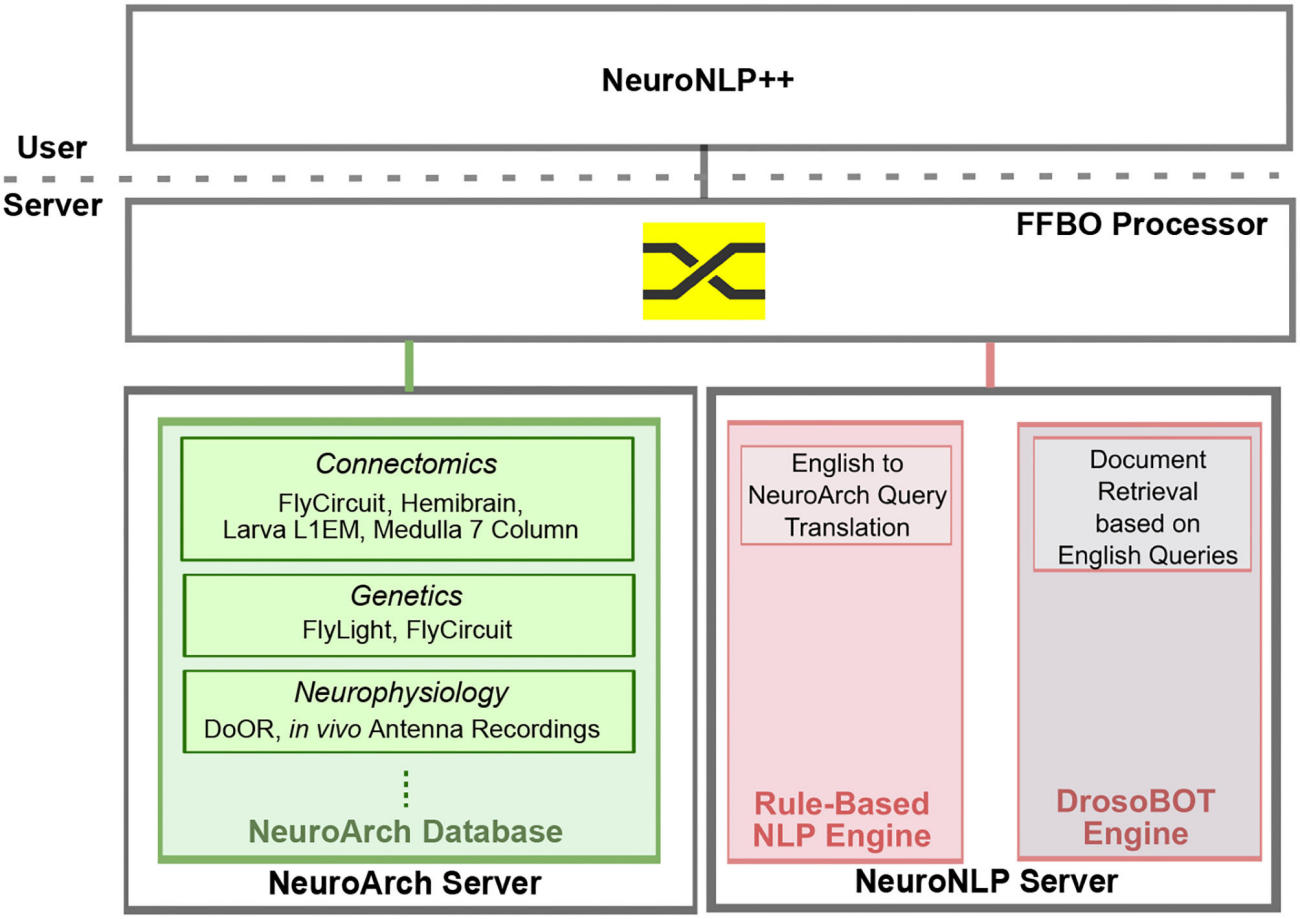
Software architecture of the NeuroNLP++ web application. The frontend application communicates with three backend servers: a NeuroArch Server that hosts a NeuroArch Database of fruit fly brain datasets, a NeuroNLP Server that runs the rule-based NLP Engine, and, an additional NeuroNLP Server that runs the DrosoBOT Engine.

NeuroNLP is a web application that supports the exploration of fruit fly brain datasets with rule-based English queries (Ukani et al., 2019; Lazar et al., 2021). To enhance the user experience when asking questions that are well beyond the current capabilities of NeuroNLP, we devised the NeuronNLP++ brain explorer (https://plusplus.neuronlp.fruitflybrain.org). Figure 12 depicts the software architecture of the NeuroNLP++ application. In addition to the backend servers supporting the NeuroNLP web application (NeuroArch Server and NeuroNLP Server with rule-based NLP Engine Ukani et al., 2019; Lazar et al., 2021), NeuroNLP++ is supported by the DrosoBOT Engine (see below), i.e., an additional backend of the NeuroNLP Server.

A free-form English query submitted through NeuroNLP++ that cannot be interpreted by the rule-based NLP engine is redirected to DrosoBOT. DrosoBOT responds with a list of ontological entities that are most pertinent. Each entry in the list includes the name of the cell type, a link to the *Drosophila* Anatomy Ontology containing references to the entities in question (Costa et al., 2013), and a description of the cell type as well as relevant entries to the worldwide literature (see also the NeuroNLP++ window in Figure 3A). Each entry also includes buttons to add, pin and unpin the neurons in the 3D visualization workspace. The morphology of the neurons is retrieved from the NeuroArch Database (Givon et al., 2015) via the NeuroArch Server and displayed using custom visualization in the browser (Ukani et al., 2019; Lazar et al., 2021) (see Figure 12).

In addition to using DrosoBOT to resolve free-form English queries, NeuroNLP++ includes an application called Graph View that allows users to visualize the graph representing the connectivity of neurons in their workspace at neuronal or cell type level. Once the Graph View button is pressed, NeuroNLP++ retrieves the connectivity of all the neurons in the workspace, with an additional capability to filter out the connections that have less than *N* synapses, where *N*≥0. A graph is then plotted in the workspace using the sigma.js library.

For neuronal level graph, each node represents a single neuron in the visualization workspace, and the edge between two nodes indicates a positive number of synaptic connections between the corresponding neurons. For a cell type level graph, each node represents a cell type that may abstract multiple neurons in the visualization workspace. An edge indicates that there exists at least 1 synaptic connection between neurons in the two cell types. In addition, the graph in Graph View is interactive. Hovering the mouse on a node highlights the corresponding neuron or all neurons of the same cell type in the 3D visualization. The graph can be further individually rearranged.

#### 6.1.2. The DrosoBOT Engine

DrosoBOT is a natural language processing engine that 1) parses free-form English queries pertaining to entities available in an ontological dataset (Costa et al., 2013), and 2) provides morphological data from a connectome dataset (Scheffer et al., 2020) already associated with each ontological entity.

Given a free-form English query, DrosoBOT first uses DPR (see below) to retrieve relevant passages in the query as context candidates, and then uses PubMedBERT, fine-tuned on the Stanford Question Answering Dataset, to find possible answers to questions pertaining to a collection of *Drosophila*-specific ontology terms and their descriptions. Here DPR is the dense passage retriever trained on the Natural Questions dataset (Kwiatkowski et al., 2019) that uses real anonymized queries issued to Google and annotated answers from the top 5 Wikipedia articles. PubMedBERT is the Bidirectional Encoder Representations from Transformers (BERT) (Devlin et al., 2018) model with biomedical domain-specific pre-training (Gu et al., 2021) from abstracts on PubMed.

In addition, for specific cell types, DrosoBOT implements a modular lexical search subsystem that uses domain knowledge to improve search results when specific keywords of cell types are asked. We make use of this system to improve the search results for the Antennal Lobe, which requires biological nomenclatures such as “DM4” to be detected not as typos but as important structures.

To bridge the gap between existing ontology and connectome datasets, we associated with each ontological entity the corresponding neurons in the Hemibrain dataset based on the names of the entities and their synonyms after searching through all possible matches in the *Drosophila* Anatomy Ontology (DAO) dataset (Costa et al., 2013). We then created a graph with nodes consisting of both names of entities in the DAO and names of neurons. An edge is created between two nodes with a matching term. After finding the ontological term relevant to the English query from the first step, we then retrieved the names of the neurons that are the graph neighbors of the ontological entity, and finally retrieved the neurons from the database.

For the AL, starting with the terms for cell types and abstractions in Costa et al. (2013) and expanding these to include references to all cell types so that all common synonyms are accounted for (for example, PNs, OSNs, glomeruli and LNs), we facilitated the specification of Antennal Lobe circuits through queries. Here we provided the capability to add relevant groups of neurons such as new glomeruli and local neurons in only a few searches and button presses. We also added the names of all glomeruli as special “keywords" whose association with the Antennal Lobe is automatically detected if present in a search query.

#### 6.1.3. Morphology and Graph Abstractions of Cell Types in the Antennal Lobe

The morphology of OSNs, PNs and LNs is retrieved, as shown in Figure 4, by using NeuroNLP++. Graph abstractions of OSNs and PNs are obtained by invoking the cell type level algorithm of Graph View.

The full list of morphological LN-types in the Antennal Lobe is presented in Supplementary Figure S1 in Supplementary Material. Each row depicts an instance of LN-type, the name of the LN-type as defined in the Hemibrain dataset, and the number of instances of LN-types. The connectivity of the shown instances of LNs with OSN and PNs arborizing in each of the 51 olfactory glomeruli is represented as a matrix on the right. The matrix entries are the number of synaptic connections of the LNs from/to OSNs and PNs. Here, we employed custom code to retrieve synaptome information directly from the NeuroArch Database.

#### 6.1.4. Morphology and Graph Abstractions of Feedback Circuits in the Antennal Lobe

To identify feedback loops in the AL, we extracted from the Hemibrain dataset (stored in the NeuroArch Database) the number of synapses between each LN-type and each type of OSNs and PNs. Here, we only considered a synapse if both its presynaptic and postsynaptic sites are identified at a confidence level higher than 70%. An LN forms an OSN-LN-OSN feedback loop if it receives a total of more than 5 synapses from all the OSNs and provides a total of more than 5 synapses to all the OSNs, and it has less than 5 synapses with all the PNs. Similarly, we consider that an LN forms a PN-LN-PN feedback loop if it receives from and provides to all the PNs a total of more than 5 synapses and has less than 5 synapses with all the OSNs. The LNs identified with each type of feedback loop in a glomerulus are then associated with an ontological entity that is accessible by DrosoBOT for document retrieval.

### 6.2. Creating the Programmable Ontology of the *Drosophila* Brain

We tightly integrated the programmable ontology of the fruit fly brain into the workflow of the interactive FlyBrainLab computing platform depicted in Figure 2 (Lazar et al., 2021).

To improve the support of the visualization of the fly brain morphology, we integrated using NeuroNLP++ the *Drosophila* anatomical ontology with the connectome/synaptome data of the Hemibrain dataset. In the FlyBrainLab workflow shown in Figure 2 this integration factors into the first step of the left.

The programmable ontology provides a methodology to define abstractions of fly brain circuits and create executable circuit diagrams as described in the second step of the FlyBrainLab workflow shown in Figure 2. FlyBrainLab provides the support for the visualization and user interaction with executable circuit diagrams.

Finally, programmability of the ontology is supported by the FlyBrainLab in the third step of the workflow to configure, compose and execute neural circuit models and to evaluate their functional logic.

#### 6.2.1. Receptor-Centric Modeling of the Space of Odorants

To construct odorant objects as elements of the space of odorants, we employed the receptor-centric Odorant Transduction Process (OTP) model developed in Lazar and Yeh (2020). In steady-state, the estimated affinity tensor **b**/**d** with entries [**b**]_*ron*_/[**d**]_*ron*_, for all *r* = 1, 2, ..., *R*, *o* = 1, 2, ..., *O* and *n* = 1, 2, ..., *N*, matches the spike rate response of the OTP model with the spike rate of the neurophysiology recordings (Hallem and Carlson, 2006) obtained in response to a constant amplitude waveform of 110 different odorants. Detailed data can be found in the olfactory transduction circuit library OlfTransCircuit available at https://github.com/FlyBrainLab/OlfTrans (Lazar et al., 2021).

#### 6.2.2. Modeling/Constructing Individual Glomeruli in the Antennal Lobe Circuit

As a first step in modeling an individual glomerulus, we first extracted all OSNs with axons arborizing a glomerulus and all the PNs that innervate that glomerulus.

To abstract the connectivity patterns of the LNs leading to the circuit diagram in Figure 7, we inspected all 311 LNs in the Hemibrain dataset (Scheffer et al., 2020). Of these, 226 LNs have more than 10 synapses in the right hemisphere AL. We only considered a synapse if both its presynaptic and postsynaptic sites are identified at a confidence level higher than 70% in the Hemibrain dataset. For each of these LNs, we counted the number of synapses they make, presynaptically and postsynaptically, with partner OSNs as well as PNs in each glomerulus. If the total number of synapses within a glomerulus is less than 5, we deem the port connectivity pattern to be 0000, i.e., no connections. The first digit of the 4-digit binary code is 1 if the number of LN to OSNs synapses is larger than 5. Similarly, the second digit is 1 if the number of synapses the LN receives from OSNs is larger than 5. The third digit is 1 if the number of LN to PNs synapses is larger than 5. Similarly, the fourth digit is 1 if the number of synapses the LN receives from PNs is larger than 5.

After inspecting all 226 LNs that innervate the right AL in the hemibrain dataset (Scheffer et al., 2020), we listed in the 2nd column of Supplementary Table S1 in Supplementary Material the number of instances each port connectivity pattern occurs across 51 olfactory glomeruli. For the DM4 and DL5 glomeruli, the number of occurrences of each port connectivity pattern is listed in the 3rd and 4th column, respectively.

#### 6.2.3. Modeling/Constructing a Pair of Interconnected Glomeruli in the AL

The modeling of interconnected glomeruli starts with the initialization of isolated glomeruli as described above. LN1s and LN2s feedback motifs can then be easily composed across glomeruli. Composition of the resulting feedback loops with LN3 leads to an interconnect of two glomeruli. These are very simple composition rules that open new directions in exploring the functional logic of interconnected glomeruli.

### 6.3. Exploring the Functional Logic of the Feedback Circuits in the Antennal Lobe

#### 6.3.1. Interactively Exploring Circuit Diagrams With the FeedbackCircuits Library

The FeedbackCircuits Library mentioned earlier in Section 4.3 was developed in Python and designed to be integrated into the FlyBrainLab ecosystem for constructing feedback circuits and exploring their function.

**Figure 13:**
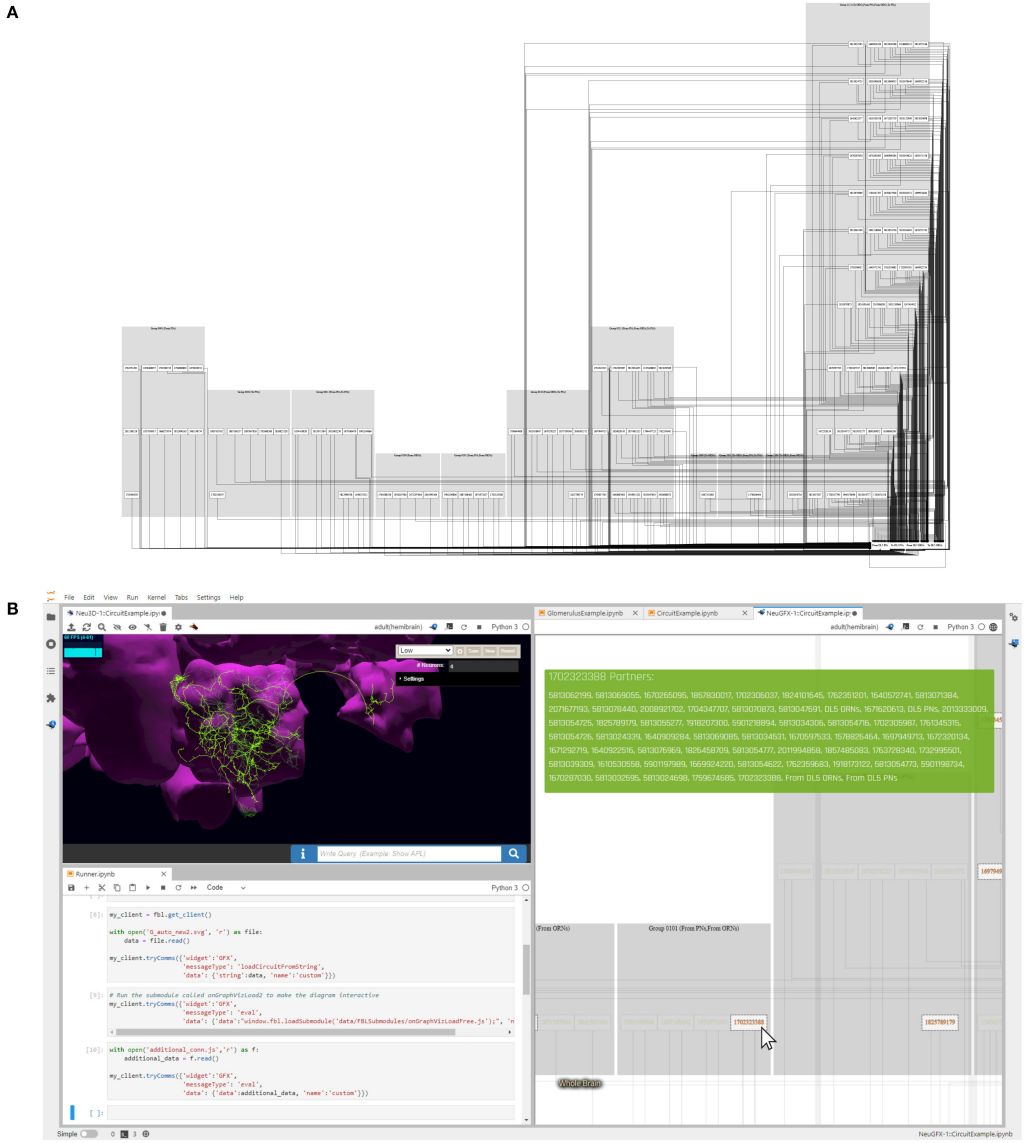
Exploring the feedback loops between the DL5 and DM4 glomeruli using an interactive circuit diagram generated by the FeedbackCircuits Library. **(A)** Users can generate a circuit diagram for any glomerulus consisting of OSNs, PNs, and LNs grouped according to the feedback connectivity pattern described in Figure 7E, such as the diagram here for the DL5 glomerulus. **(B)** The generated diagrams are interactive in the FlyBrainLab platform. Hovering over the neurons on the diagram (right) shows their partners, and highlights them in the diagram and in the corresponding 3D morphology (top left); clicking disables/enables them for program execution (bottom left). Here, the user is currently hovering over the Hemibrain neuron with identifier 1702323388, which shows its partners in the green block on the top right window and highlights it on the morphology in the top left in green.

The FeedbackCircuits Library provides tools for interactively visualizing and exploring the feedback loops of the AL model circuits that are operational on the FlyBrainLab computing platform (Lazar et al., 2021). Figure 13A depicts an automatically generated circuit diagram of the DL5 glomerulus. This circuit diagram is a schematic of the glomerulus model shown in Figure 7E and is based, for OSNs and PNs, upon the Hemibrain connectome dataset (Scheffer et al., 2020). LNs are grouped into blocks according to their port connectivity patterns with respect to a given glomerulus (here, DL5). The circuit diagram is fully operational in the FlyBrainLab platform; the interactive user interface is shown in Figure 13B. On the top left is the NeuroNLP 3D visualization window for displaying the morphology of neurons. At the bottom left a Jupyter notebook for code execution is displayed. The circuit diagram allows users to inspect the morphology of neurons in the NeuroNLP window by clicking on the ones displayed. For example, in Figure 13B (right), we zoomed into the LNs that exhibit the port connectivity pattern 0101. By clicking on the neuron whose Hemibrain ID is 1702323388, the LN in green is highlighted in Figure 13B (top left). Hovering also highlights all connected neurons, such as the one with Hemibrain ID 1825789179 in Figure 13B (right). The interactive circuit diagram provides an intuitive means of constructing feedback motifs from connectome data.

#### 6.3.2. Evaluating the Role of Feedback Circuits in a Single Glomerulus

As already briefly mentioned above, to construct the DM4 glomerulus feedback circuits with the FeedbackCircuits Library, we created a DM4 glomerulus object that includes all the OSNs and PNs and their connections according to the Hemibrain dataset. We then add feedback motifs LN1, LN2, and LN3 from Figure 8C. The construction of the DL5 glomerulus including the feedback motifs follows a similar procedure.

To model the odorant transduction process of the OSNs, we followed the OTP model in Lazar and Yeh (2020). The axon hillock of OSNs, PNs and LNs is given by the Connor- Stevens point neuron model (Connor and Stevens, 1971). All synapses are modeled as a variation of the α synapse. We also modeled the presynaptic effect of LNs onto the OSN axon terminal. A detailed description of the dynamics of the olfactory transduction process, neurons and synapses can be found in Section 2 of Supplementary Material. The configured model circuits were executed on the Neurokernel Execution Engine (Givon and Lazar, 2016). Neurokernel supports the execution of spiking and/or analog neuron models.

To evaluate the feedback motifs, we swept through all possible concentration-modulated affinity values, defined as [**b**]_*ron*_/[**d**]_*ron*_·[**u**]_*o*_, where [**b**]_*ron*_/[**d**]_*ron*_ is the affinity odorant *o* with the receptor *r* expressed by OSN *n*, and [**u**]_*o*_ is the constant concentration waveform of odorant *o* presented to OSN *n*.

#### 6.3.3. Evaluating the Role of Feedback Circuits in/Between a Pair of Glomeruli

To construct the feedback circuit interconnecting a pair of glomeruli (e.g., DM4 and DL5), we started with two independent circuits, with feedback motifs LN1 and LN2, that only innervate a single glomerulus. We then composed these two independent circuits across the two glomeruli, and added a feedback motif LN3 that connects to each LN1 and LN2 in both directions. Instead of stimulating LN3 externally, we assumed that synapses from LN1 and LN2 to LN3 are excitatory, and the output of LN3 is inhibitory.

The olfactory transduction, axon hillock and synaptic models of the interconnected pair of glomeruli are the same as the ones of the single glomerulus above, and their dynamics are described in detail in Section 2 of Supplementary Material.

To evaluate the feedback circuit of the pair of interconnected glomeruli, we swept through constant inputs on a grid of concentration-modulated affinity values associated with the odorant receptor of OSNs with axons that arborize the DM4 and DL5 glomeruli, respectively. PN responses to the inputs with values on lines crossing the origin can be used to characterize the responses to the odorants of interest.

#### 6.3.4. Modeling and Constructing the Massive Feedback Circuits of the AL

Composition of the circuit diagram of the entire AL in Figure 11 comes in three steps. First, OSNs and PNs and their ports from/to LNs are constructed for each glomerulus according to Figure 7, where the glomeruli are depicted as cylinders at the bottom of Figure 11B. Second, for each glomerulus, we also configure the connectivity patterns of the LNs, as described in Section 4.3. This forms the local crossbar between all LNs and the ports of a glomerulus and is depicted, e.g., on the top right of Figure 11B, as 4 vertical lines. Finally, by connecting the local crossbars from all glomeruli with the innervation pattern of each LN, we obtain a hierarchical crossbar between LNs and the ports of the glomeruli. The hierarchical crossbar provides the flexibility to configure the routing of interconnections across glomeruli, either by using the port connectivity patterns of LNs extracted from connectome data (see also Supplementary Figure S1 in Supplementary Material), or by any variations/ablations thereof for testing and evaluating the functional logic of the AL circuit.

## Code Availability Statement

NeuroNLP++ web application is available at https://plusplus.neuronlp.fruitflybrain.org. It is also available as a Docker machine image for standalone installations. The FeedbackCircuits Library is available as a Python package at https://github.com/mkturkcan/FeedbackCircuits. The FeedbackCircuits Library includes a number of example Jupyter notebooks that help users explore its functionality. For installation of FlyBrainLab, refer to Lazar et al. (2021).

## Data Availability Statement

Publicly available datasets were analyzed in this study. This data can be found here: https://www.fruitflybrain.org. The NeuroArch Database hosting publicly available Hemibrain dataset that are used in the exploration and validation in this paper can be downloaded from https://github.com/FlyBrainLab/datasets.

## Author Contributions

AL contributed to conceptualization, developing research directions, investigation, methodology, writing—original draft, writing—review and editing, resources, supervision, funding acquisition, and project administration. MT contributed to conceptualization, software, validation, investigation, visualization, methodology, writing—original draft, writing—review and editing. Developed NeuroNLP++ and the FeedbackCircuits library. Performed evaluation of the AL feedback circuit. YZ contributed to conceptualization, developing research directions, validation, investigation, visualization, methodology, writing—original draft, writing—review and editing, funding acquisition, and project administration. All authors contributed to the article and approved the submitted version.

## Funding

The research reported here was supported by AFOSR under grant #FA9550-16-1-0410, DARPA under contract #HR0011-19-9-0035 and NSF under grant #2024607.

## Conflict of Interest

The authors declare that the research was conducted in the absence of any commercial or financial relationships that could be construed as a potential conflict of interest.

## Publisher's Note

All claims expressed in this article are solely those of the authors and do not necessarily represent those of their affiliated organizations, or those of the publisher, the editors and the reviewers. Any product that may be evaluated in this article, or claim that may be made by its manufacturer, is not guaranteed or endorsed by the publisher.

## References

[B1] Bakken T. E. Jorstad N. L. Hu Q. Lake B. B. Tian W. Kalmbach B. E.. (2021). Comparative cellular analysis of motor cortex in human, marmoset and mouse. Nature 598, 111–119. 10.1038/s41586-021-03465-8 34616062 PMC8494640

[B2] Borst A. Haag J. Mauss A. S. (2020). How fly neurons compute the direction of visual motion. J. Compar. Physiol. A 206, 109–124. 10.1007/s00359-019-01375-9 31691093 PMC7069908

[B3] Chiang A.-S. Lin C.-Y. Chuang C.-C. Chang H.-M. Hsieh C.-H. Yeh C.-W.. (2011). Three-dimensional reconstruction of brain-wide wiring networks in *Drosophila* at single-cell resolution. Curr. Biol. 21, 1–11. 10.1016/j.cub.2010.11.056 21129968

[B4] Chou Y.-H. Spletter M. L. Yaksi E. Leong J. C. S. Wilson R. I. Luo L. (2010). Diversity and wiring variability of olfactory local interneurons in the *Drosophila* antennal lobe. Nat. Neurosci. 13, 439–449. 10.1038/nn.2489 20139975 PMC2847188

[B5] Clements J. Dolafi T. Umayam L. Neubarth N. L. Berg S. Scheffer L. K.. (2020). neuprint: analysis tools for em connectomics. bioRxiv. 10.1101/2020.01.16.909465 PMC9350508 35935535

[B6] Connor J. A. Stevens C. F. (1971). Prediction of repetitive firing behavior from voltage clamp data on an isolated neurone soma. J. Physiol. 213, 31–53. 10.1113/jphysiol.1971.sp009366 5575343 PMC1331721

[B7] Costa M. Reeve S. Grumbling G. Osumi-Sutherland D. (2013). The *Drosophila* anatomy ontology. J. Biomed. Semantics 4, 32. 10.1186/2041-1480-4-32 24139062 PMC4015547

[B8] Devlin J. Chang M.-W. Lee K. Toutanova K. (2018). Bert: pre-training of deep bidirectional transformers for language understanding. arXiv preprint arXiv:1810.04805. 10.48550/arXiv.1810.0480535689168

[B9] Egelhaaf M. Kern R. Krapp H. G. Kretzberg J. Kurtz R. Warzecha A.-K. (2002). Neural encoding of behaviorally relevant visual-motion information in the fly. Trends Neurosci. 25, 96–102. 10.1016/S0166-2236(02)02063-5 11814562

[B10] Eschbach C. Fushiki A. Winding M. Schneider-Mizell C. M. Shao M. Arruda R.. (2020). Recurrent architecture for adaptive regulation of learning in the insect brain. Nat. Neurosci. 23, 544–555. 10.1038/s41593-020-0607-9 32203499 PMC7145459

[B11] Givon L. E. Lazar A. A. (2016). Neurokernel: an open source platform for emulating the fruit fly brain. PLoS ONE 11, e0146581. 10.1371/journal.pone.0146581 26751378 PMC4709234

[B12] Givon L. E. Lazar A. A. Ukani N. H. (2015). Neuroarch: A graph db for querying and executing fruit fly brain circuits. Zenodo. 10.5281/zenodo.44225

[B13] Givon L. E. Lazar A. A. Yeh C.-H. (2017). Generating executable models of the *Drosophila* central complex. Front. Behav. Neurosci. 11, 102. 10.3389/fnbeh.2017.00102 28611607 PMC5447672

[B14] Grünert U. Martin P. R. (2020). Cell types and cell circuits in human and non-human primate retina. Prog. Retin Eye Res. 78, 100844. 10.1016/j.preteyeres.2020.100844 32032773

[B15] Gu Y. Tinn R. Cheng H. Lucas M. Usuyama N. Liu X.. (2021). Domain-specific language model pretraining for biomedical natural language processing. ACM Trans. Comput. Healthcare 3, 1–23. 10.1145/3458754

[B16] Hallem E. A. Carlson J. R. (2006). Coding of odors by a receptor repertoire. Cell 125, 143–160. 10.1016/j.cell.2006.01.050 16615896

[B17] Harris J. A. Mihalas S. Hirokawa K. E. Whitesell J. D. Choi H. Bernard A.. (2019). Hierarchical organization of cortical and thalamic connectivity. Nature 575, 195–202. 10.1038/s41586-019-1716-z 31666704 PMC8433044

[B18] Huang Y.-C. Wang C.-T. Su T.-S. Kao K.-W. Lin Y.-J. Chuang C.-C.. (2019). A single-cell level and connectome-derived computational model of the *Drosophila* brain. Front. Neuroinform. 12, 99. 10.3389/fninf.2018.00099 30687056 PMC6335393

[B19] Hulse B. K. Haberkern H. Franconville R. Turner-Evans D. B. Takemura S.-,y. Wolff T.. (2021). A connectome of the *Drosophila* central complex reveals network motifs suitable for flexible navigation and context-dependent action selection. Elife 10, e66039. 10.7554/eLife.66039 34696823 PMC9477501

[B20] Jeanne J. M. Fişek M. Wilson R. I. (2018). The organization of projections from olfactory glomeruli onto higher-order neurons. Neuron 98, 1198.e6–1213.e6. 10.1016/j.neuron.2018.05.011 29909998 PMC6051339

[B21] Karpukhin V. Oğuz B. Min S. Lewis P. Wu L. Edunov S.. (2020). Dense passage retrieval for open-domain question answering. arXiv preprint arXiv:2004.04906. 10.18653/v1/2020.emnlp-main.55035634104

[B22] Kwiatkowski T. Palomaki J. Redfield O. Collins M. Parikh A. Alberti C.. (2019). Natural questions: a benchmark for question answering research. Trans. Assoc. Comput. Linguist. 7, 453–466. 10.1162/tacl_a_00276

[B23] Lamme V. A. Supèr H. Spekreijse H. (1998). Feedforward, horizontal, and feedback processing in the visual cortex. Curr. Opin. Neurobiol. 8, 529–535. 10.1016/S0959-4388(98)80042-1 9751656

[B24] Lazar A. A. Liu T. Turkcan M. K. Zhou Y. (2021). Accelerating with flybrainlab the discovery of the functional logic of the *Drosophila* brain in the connectomic and synaptomic era. Elife 10, e62362. 10.7554/eLife.62362 33616035 PMC8016480

[B25] Lazar A. A. Liu T. Yeh C.-H. (2020). “An odorant encoding machine for sampling, reconstruction and robust representation of odorant identity,” in ICASSP 2020-2020 IEEE International Conference on Acoustics, Speech and Signal Processing (ICASSP) (Barcelona: IEEE), 1743–1747.

[B26] Lazar A. A. Liu T. Yeh C.-H. (2022). The functional logic of odor information processing in the *Drosophila* antennal lobe. bioRxiv. 10.1101/2021.12.27.474306 PMC10156017 37083547

[B27] Lazar A. A. Ukani N. H. Psychas K. Zhou Y. (2015). A parallel processing model of the *Drosophila* retina. Zenodo. 10.5281/zenodo.30036

[B28] Lazar A. A. Yeh C.-H. (2020). A molecular odorant transduction model and the complexity of spatio-temporal encoding in the *Drosophila* antenna. PLoS Comput. Biol. 16, e1007751. 10.1371/journal.pcbi.1007751 32287275 PMC7182276

[B29] Li H. Janssens J. De Waegeneer M. Kolluru S. S. Davie K. Gardeux V.. (2021). Fly cell atlas: a single-cell transcriptomic atlas of the adult fruit fly. bioRxiv. 10.1101/2021.07.04.451050 35239393 PMC8944923

[B30] Modi M. N. Shuai Y. Turner G. C. (2020). The *Drosophila* mushroom body: from architecture to algorithm in a learning circuit. Ann. Rev. Neurosci. 43, 465–484. 10.1146/annurev-neuro-080317-0621333 32283995

[B31] Morgan H. L. (1965). The generation of a unique machine description for chemical structures-a technique developed at chemical abstracts service. J. Chem. Doc. 5, 107–113. 10.1021/c160017a018

[B32] Nern A. Pfeiffer B. D. Rubin G. M. (2015). Optimized tools for multicolor stochastic labeling reveal diverse stereotyped cell arrangements in the fly visual system. Proc. Natl. Acad. Sci. U.S.A. 112, E2967–E2976. 10.1073/pnas.1506763112 25964354 PMC4460454

[B33] Ohyama T. Schneider-Mizell C. M. Fetter R. D. Aleman J. V. Franconville R. Rivera-Alba M.. (2015). A multilevel multimodal circuit enhances action selection in Drosophila. Nature 520, 633–639. 10.1038/nature14297 25896325

[B34] Paxions G. Watson C. (2013). The Rat Brain in Stereotaxic Coordinates. Elsevier Academic Press. Available online at: https://www.elsevier.com/books/the-rat-brain-in-stereotaxic-coordinates/paxinos/978-0-12-391949-6

[B35] Poldrack R. A. Yarkoni T. (2016). From brain maps to cognitive ontologies: informatics and the search for mental structure. Ann. Rev. Psychol. 67, 587–612. 10.1146/annurev-psych-122414-033729 26393866 PMC4701616

[B36] Scheffer L. K. Xu C. S. Januszewski M. Lu Z. Takemura S.-,y. Hayworth K. J.. (2020). A connectome and analysis of the adult *Drosophila* central brain. Elife 9, e57443. 10.7554/eLife.57443 32880371 PMC7546738

[B37] Scott C. A. Dahanukar A. (2014). Sensory coding of olfaction and taste. Behav. Genet. Fly 2, 49. 10.1017/CBO9780511920585.005

[B38] Shapson-Coe A. Januszewski M. Berger D. R. Pope A. Wu Y. Blakely T.. (2021). A connectomic study of a petascale fragment of human cerebral cortex. bioRxiv. 10.1101/2021.05.29.446289

[B39] Sunkin S. M. Ng L. Lau C. Dolbeare T. Gilbert T. L. Thompson C. L.. (2012). Allen brain atlas: an integrated spatio-temporal portal for exploring the central nervous system. Nucleic Acids Res. 41, D996–D1008. 10.1093/nar/gks1042 23193282 PMC3531093

[B40] Swanson L. W. (2018). Brain maps 4.0–structure of the rat brain: An open access atlas with global nervous system nomenclature ontology and flatmaps. J. Compar. Neurol. 526, 935–943. 10.1002/cne.24381 29277900 PMC5851017

[B41] Takemura S.-y. Xu C. S. Lu Z. Rivlin P. K. Parag T. Olbris D. J.. (2015). Synaptic circuits and their variations within different columns in the visual system of *Drosophila. Proc. Natl. Acad. Sci. U.S.A*. 112, 13711–13716. 10.1073/pnas.1509820112 26483464 PMC4640747

[B42] Tasic B. Yao Z. Graybuck L. T. Smith K. A. Nguyen T. N. Bertagnolli D.. (2018). Shared and distinct transcriptomic cell types across neocortical areas. Nature 563, 72–78. 10.1038/s41586-018-0654-5 30382198 PMC6456269

[B43] Tootoonian S. Coen P. Kawai R. Murthy M. (2012). Neural representations of courtship song in the *Drosophila* brain. J. Neurosci. 32, 787–798. 10.1523/JNEUROSCI.5104-11.2012 22262877 PMC3520490

[B44] Tran N. Kepple D. Shuvaev S. Koulakov A. (2019). “Deepnose: Using artificial neural networks to represent the space of odorants,” in Proceedings of the 36th International Conference on Machine Learning, Vol. 97 (PMLR), 6305–6314. Available online at: http://proceedings.mlr.press/v97/tran19b/tran19b.pdf

[B45] Tuthill J. C. Wilson R. I. (2016). Mechanosensation and adaptive motor control in insects. Curr. Biol. 26, R1022–R1038. 10.1016/j.cub.2016.06.070 27780045 PMC5120761

[B46] Ukani N. H. Yeh C.-H. Tomkins A. Zhou Y. Florescu D. Ortiz C. L.. (2019). The fruit fly brain observatory: from structure to function. bioRxiv. 10.1101/580290

[B47] Wang Q. Ding S.-L. Li Y. Royall J. Feng D. Lesnar P.. (2020). The allen mouse brain common coordinate framework: a 3d reference atlas. Cell 181, 936.e20–953.e20. 10.1016/j.cell.2020.04.007 32386544 PMC8152789

[B48] Yang H. H. Clandinin T. R. (2018). Elementary motion detection in *Drosophila*: algorithms and mechanisms. Ann. Rev. Vis. Sci. 4, 143–163. 10.1146/annurev-vision-091517-034153 29949723 PMC8097889

[B49] Zheng Z. Lauritzen J. S. Perlman E. Robinson C. G. Nichols M. Milkie D.. (2018). A complete electron microscopy volume of the brain of adult *Drosophila melanogaster*. Cell 174, 730.e22–743.e22. 10.1016/j.cell.2018.06.019 30033368 PMC6063995

