# Supplementary material for: A Programmable Ontology Encompassing the Functional Logic of the Drosophila Brain
Source: Front Neuroinform. 2022 Jun 20;16:853098. doi: 10.3389/fninf.2022.853098 (PMC9252271; doi:10.3389/fninf.2022.853098)
Supplement: Supplementary file 1 [file Data_Sheet_1.pdf]

Supplementary Material

1 ANTENNAL LOBE LOCAL NEURON-TYPES

In Figure S1 the full list of morphological LN-types are presented. Each row depicts an instance of the LN-type, the name of the LN-type as defined in the Hemibrain dataset, the number of instances of the LN-types. The connectivity of the instance of LN with OSN and PNs arborizing in each of the 50 glomeruli is represented as a matrix on the right.

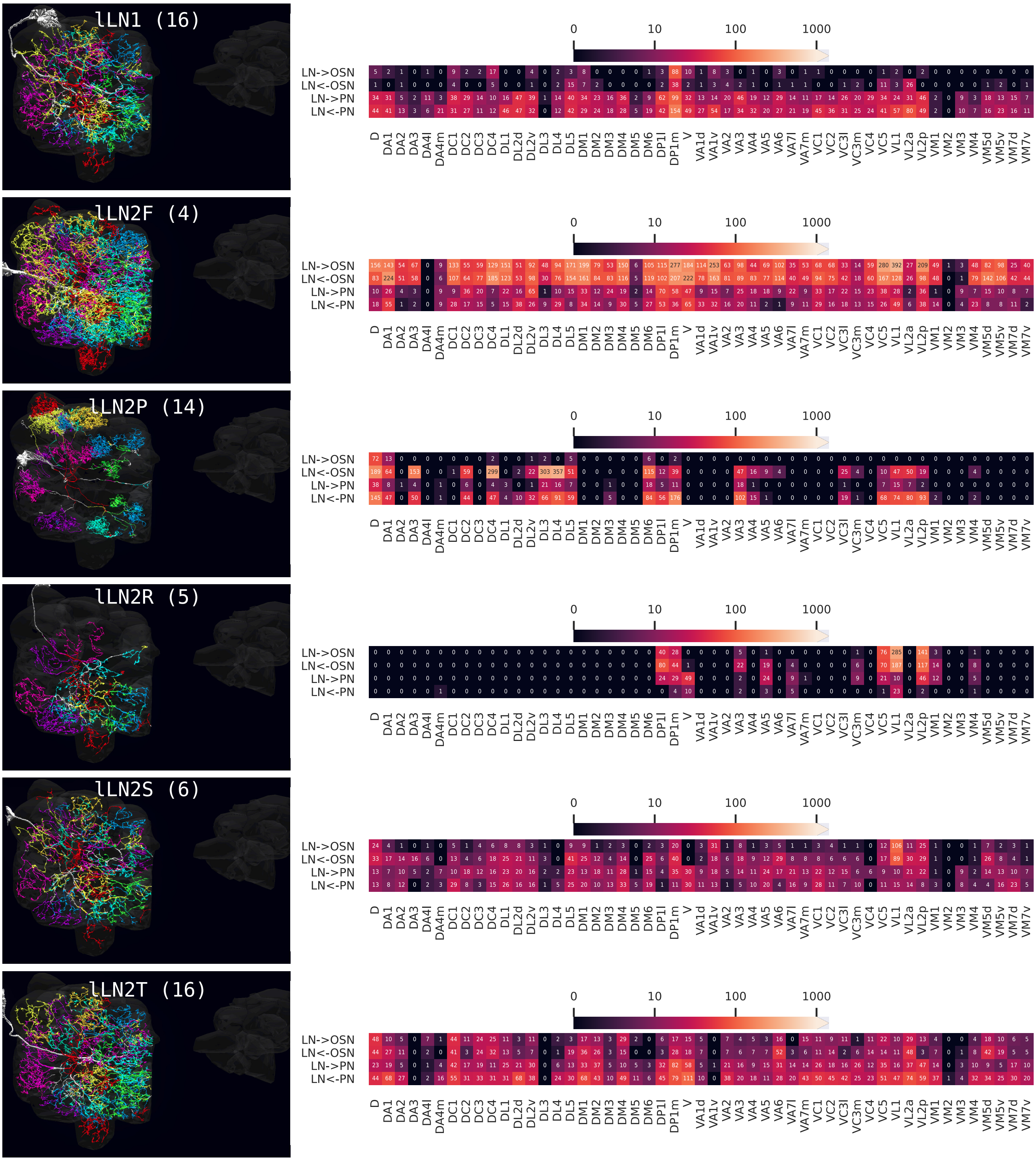

Figure S1: Morphological LN-types in the Antennal Lobe. Each row: (left) morphology of an instance of a cell type, with the integer in parenthesis indicating the number of neurons of the same cell type. Each color indicates a glomerulus that the LN arborizes. (right) The number of synapses from the LN to the OSNs in each of the 51 olfactory glomeruli (top row); the number of synapses the LN receives from the OSNs in each of the 51 olfactory glomeruli (second row); The number of synapses from the LN to the PNs in each of the 51 olfactory glomeruli (third row); the number of synapses the LN receives from the PNs in each of the 51 olfactory glomeruli (last row).

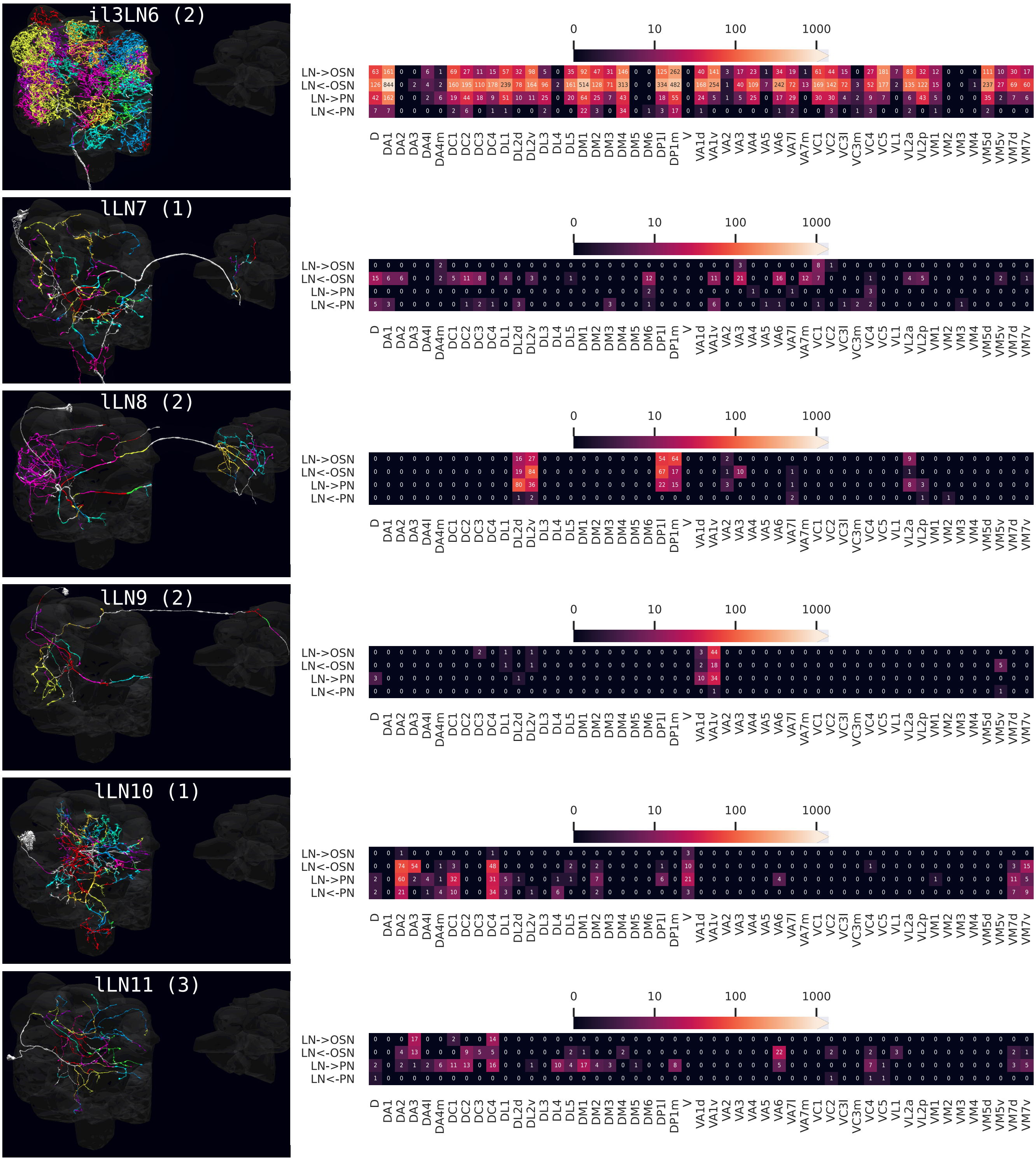

Figure S1 (cont.): Morphological LN-types in the Antennal Lobe.

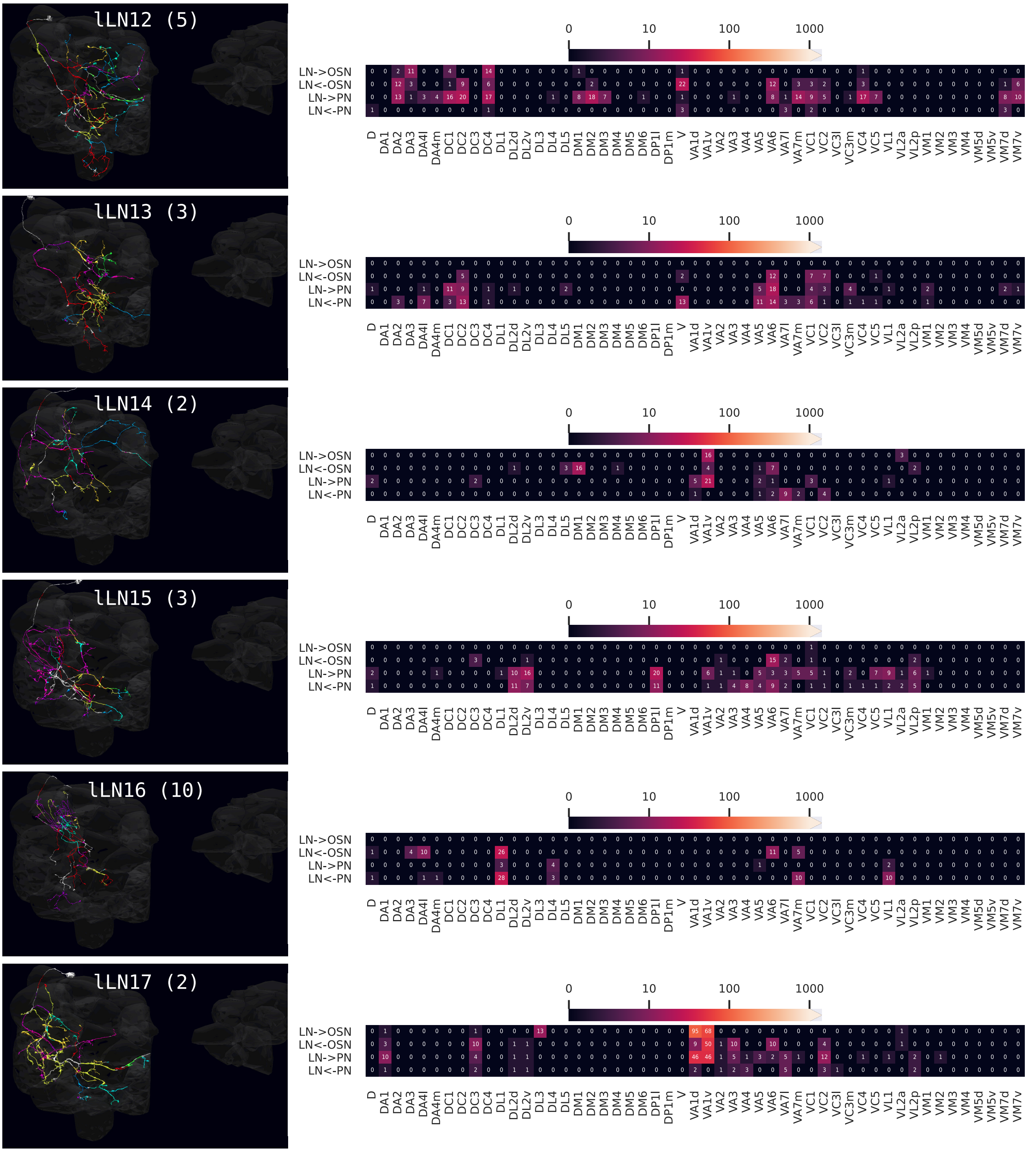

Figure S1 (cont.): Morphological LN-types in the Antennal Lobe.

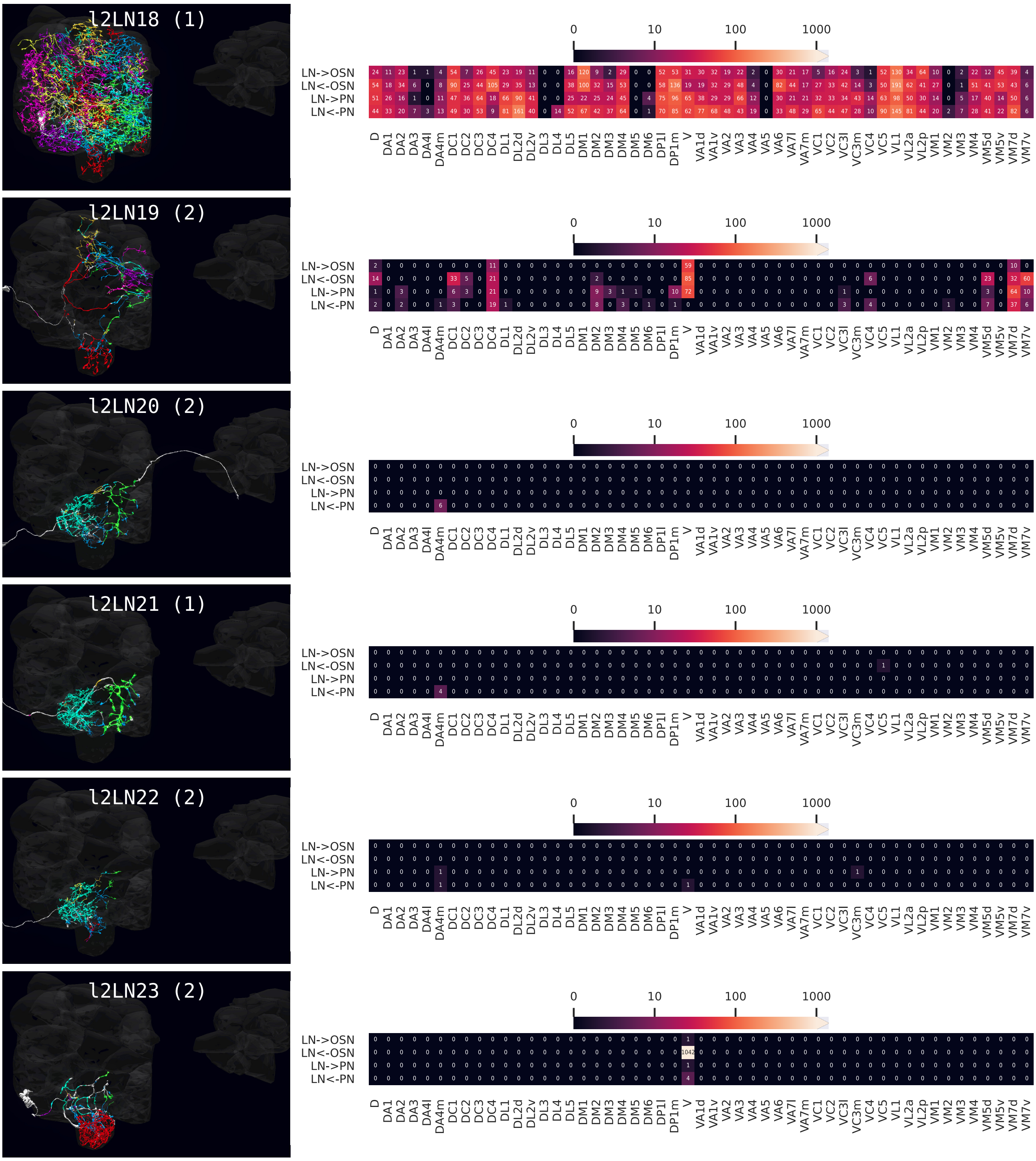

Figure S1 (cont.): Morphological LN-types in the Antennal Lobe.

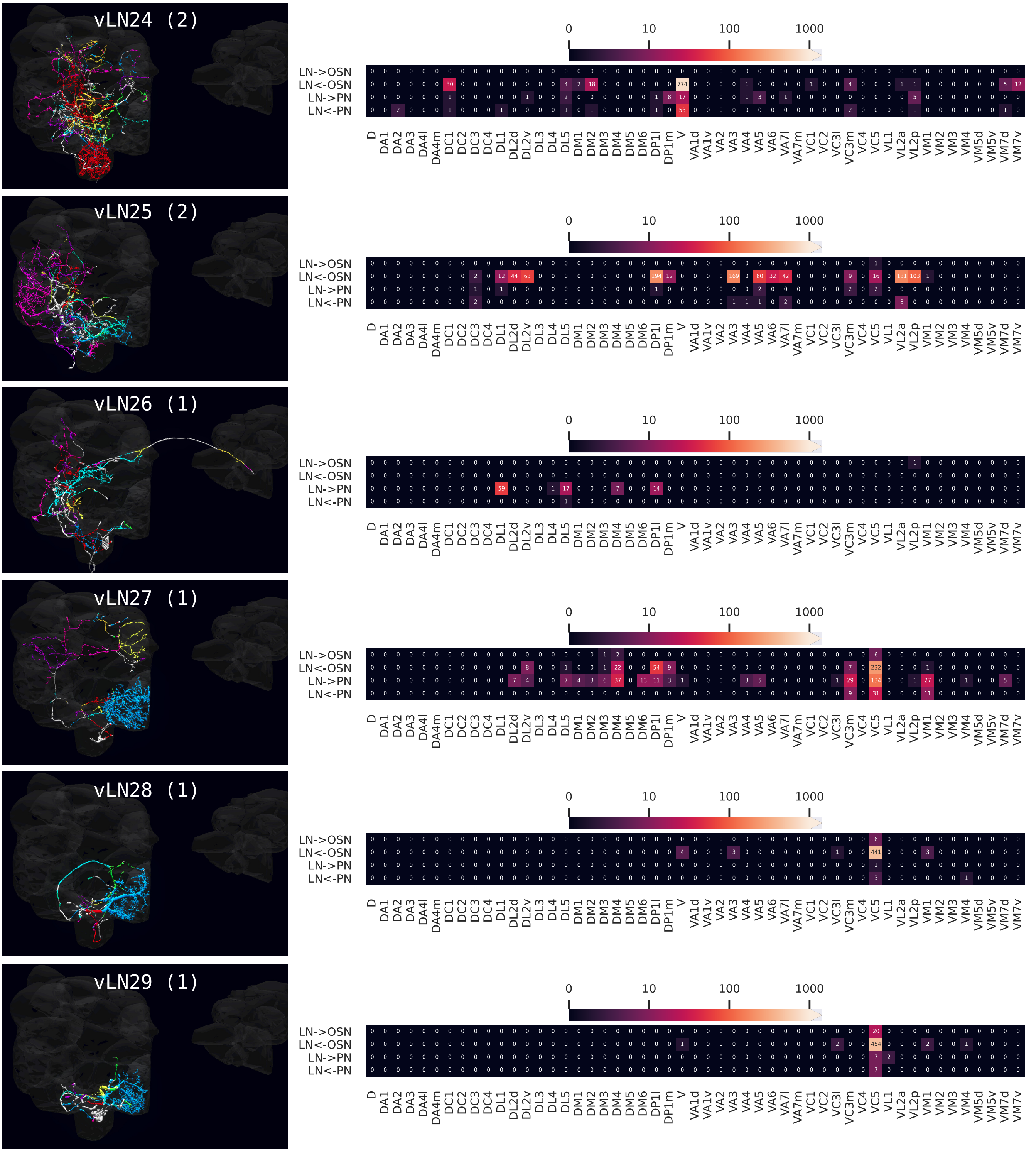

Figure S1 (cont.): Morphological LN-types in the Antennal Lobe.

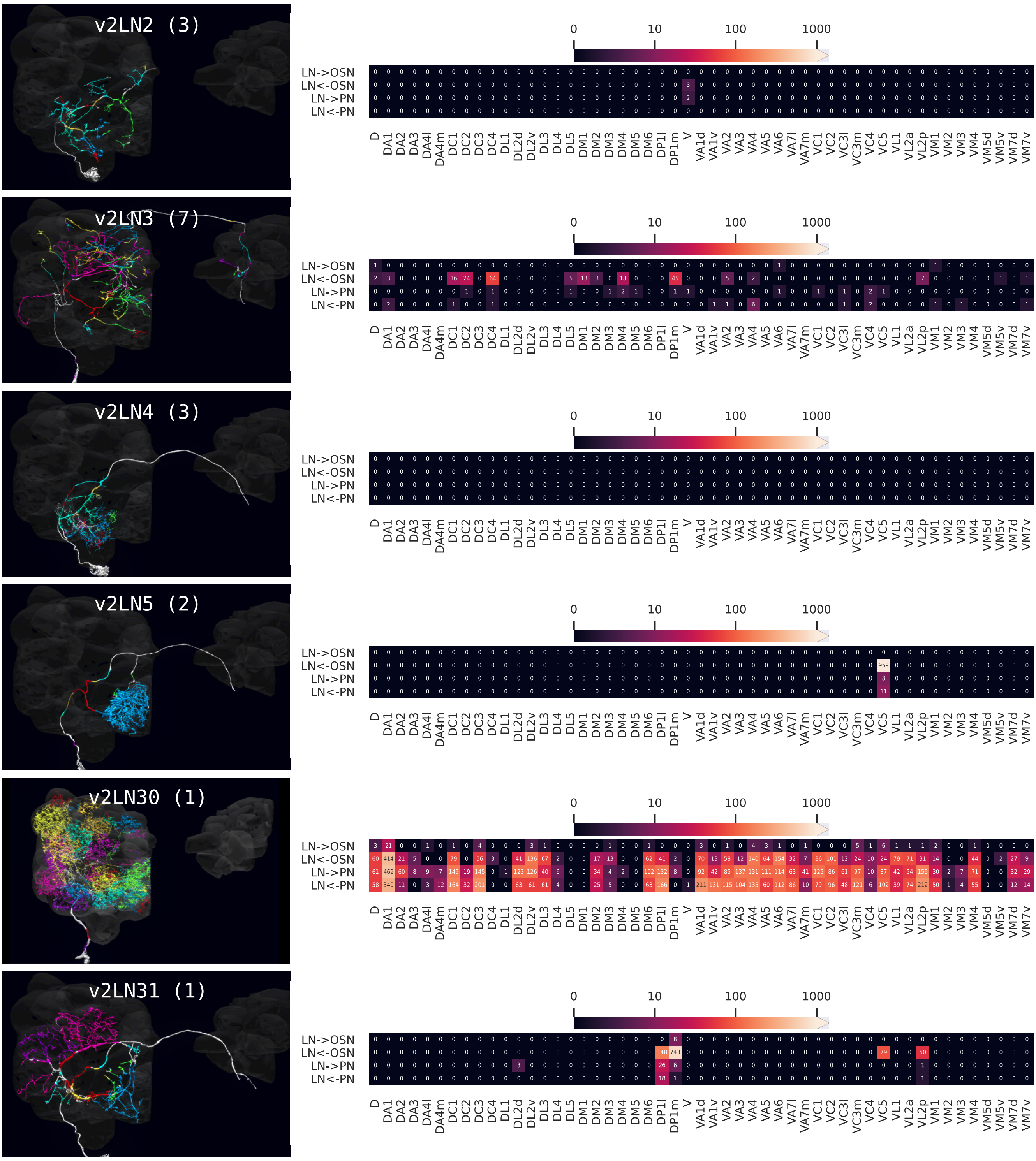

Figure S1 (cont.): Morphological LN-types in the Antennal Lobe.

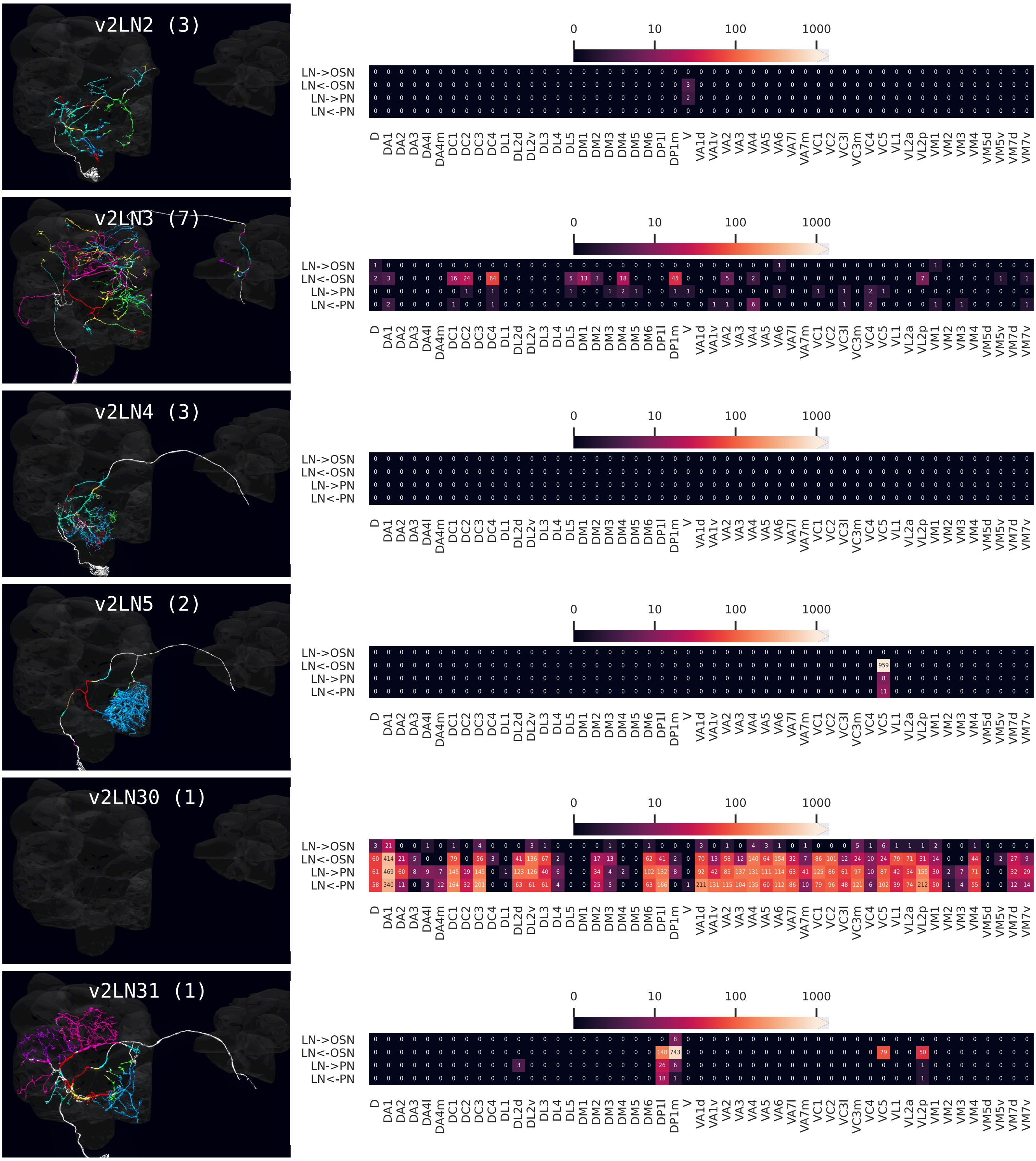

Figure S1 (cont.): Morphological LN-types in the Antennal Lobe.

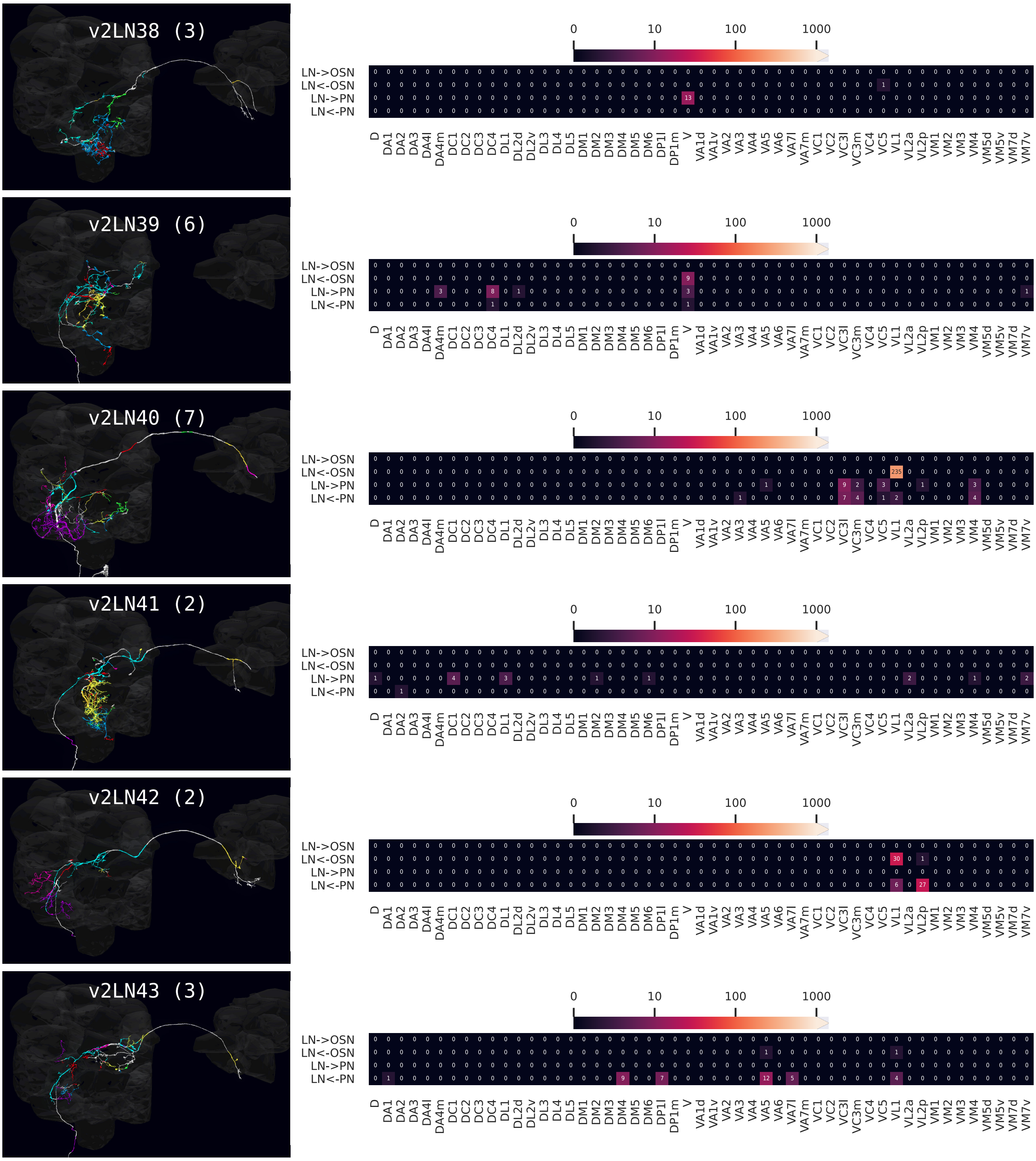

Figure S1 (cont.): Morphological LN-types in the Antennal Lobe.

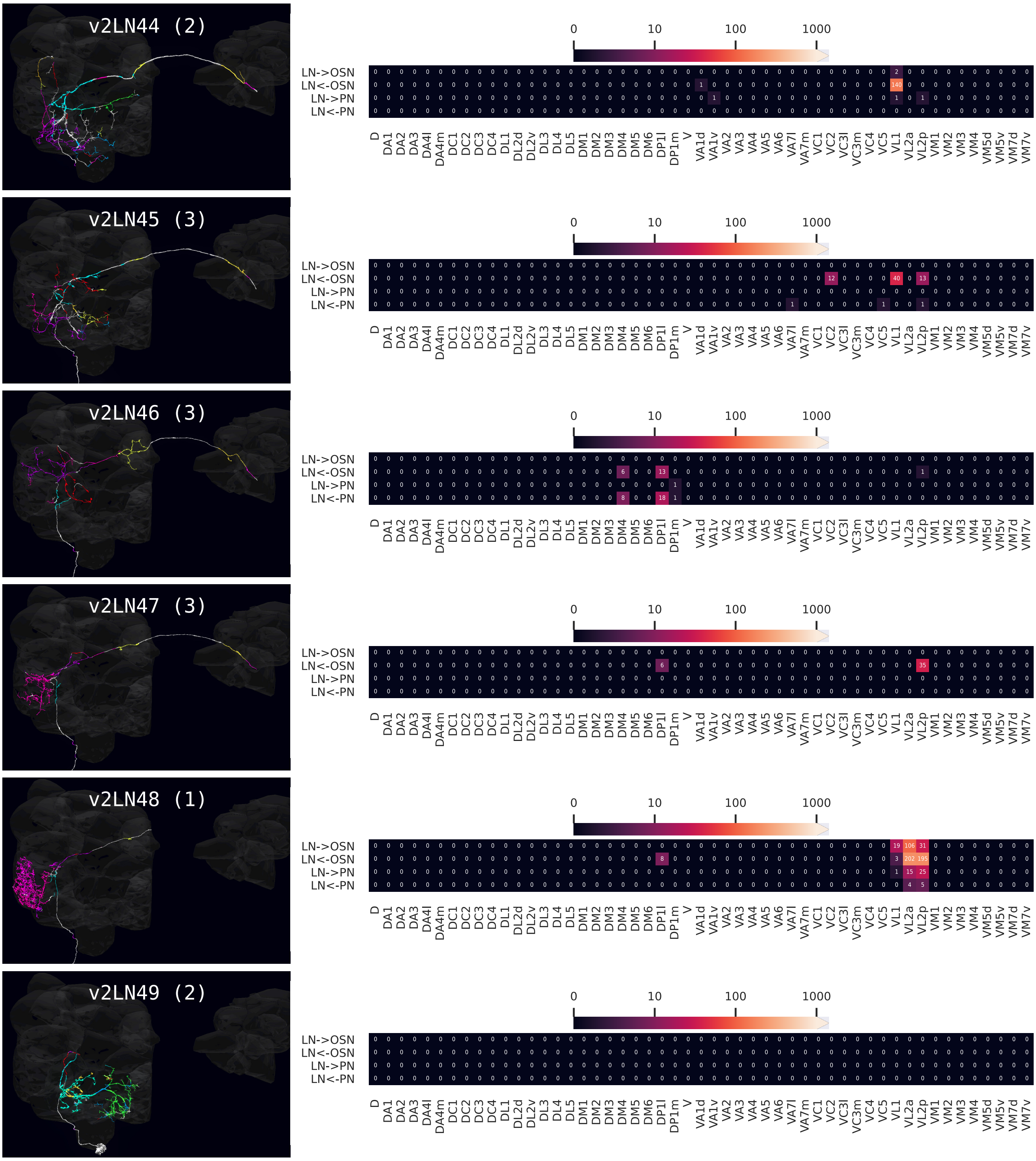

Figure S1 (cont.): Morphological LN-types in the Antennal Lobe.

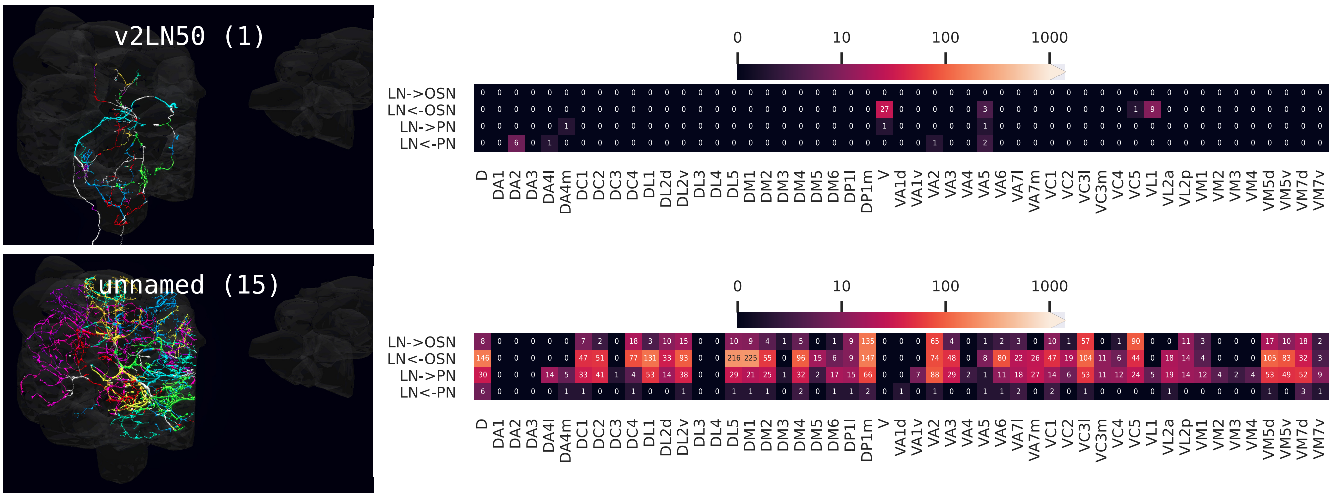

Figure S1 (cont.): Morphological LN-types in the Antennal Lobe.

2 NEURONAL/SYNAPTIC DYNAMICS AND ODORANT TRANSDUCTION PROCESS MODELS

We used the Connor-Stevens neuron model (Connor and Stevens, 1971) whose dynamics can be expressed by the system of differential equations

$$\begin{aligned} C\frac{dV}{dt} =& - \bar{g}_{Na} m^3 h (V - E_{Na}) - \bar{g}_K n^4 (V - E_K) \\ & - \bar{g}_A A^3 B (V - E_A) - g_{Cl}(V - E_{Cl}) + I_{syn}, \end{aligned} \tag{S1}$$

$$\frac{dn}{dt} = \frac{n_\infty(V) - n}{\tau_n(V)}, \tag{S2}$$

$$\frac{dm}{dt} = \frac{m_\infty(V) - m}{\tau_m(V)}, \tag{S3}$$

$$\frac{dh}{dt} = \frac{h_\infty(V) - n}{\tau_h(V)}, \tag{S4}$$

$$\frac{dA}{dt} = \frac{A_\infty(V) - A}{\tau_A(V)}, \tag{S5}$$

$$\frac{dB}{dt} = \frac{B_\infty(V) - B}{\tau_B(V)}, \tag{S6}$$

where

$$a_n(V) = \frac{-0.01(V + 50 + k_n)}{\exp\left(-\frac{V+50+k_n}{10}\right) - 1}, \quad b_n(V) = 0.125 \exp\left(-\frac{V + 60 + k_n}{80}\right), \quad n_\infty(V) = \frac{a_n(V)}{a_n(V) + b_n(V)}, \tag{S7}$$

$$a_m(V) = \frac{-0.1(V + 35 + k_m)}{\exp\left(-\frac{V+35+k_m}{10}\right) - 1}, \quad b_m(V) = 4 \exp\left(-\frac{V + 60 + k_m}{18}\right), \quad m_\infty(V) = \frac{a_m(V)}{a_m(V) + b_m(V)}, \tag{S8}$$

$$a_h(V) = 0.07\text{exp}\left(-\frac{V + 60 + k_h}{20}\right), \quad b_h(V) = \frac{1}{\exp\left(-\frac{V+30+k_h}{10}\right)}, \quad h_\infty(V) = \frac{a_h(V)}{a_h(V) + b_h(V)}, \tag{S9}$$

$$\tau_n(V) = \frac{2}{3.8(a_m(V) + b_m(V))}, \quad \tau_m(V) = \frac{1}{3.8(a_m(V) + b_m(V))}, \quad \tau_h(V) = \frac{1}{3.8(a_h(V) + b_h(V))} \tag{S10}$$

$$A_\infty(V) = \left[0.0761 \frac{\exp\left(\frac{V+94.22}{31.84}\right)}{\left(1 + \exp\left(\frac{V+1.17}{28.93}\right)\right)}\right]^{\frac{1}{3}}, \quad \tau_A = 0.3632 + \frac{1.158}{1 + \exp\left((V + 55.96)/20.12\right)}, \tag{S11}$$

$$B_\infty(V) = \frac{1}{\left(1 + \exp\left(\frac{V+53.3}{14.54}\right)\right)^4}, \quad \tau_B(V) = \left(1.24 + \frac{2.678}{1 + \exp\left(\frac{V+50}{16.027}\right)}\right), \tag{S12}$$

where V is the membrane potential, C is the capacitance, $\bar{g}$ is the maximum conductance, E denotes reversal potential, n, m, h, A and B are dimensionless quantities between 0 and 1 associated with potassium channel activation, sodium channel activation, sodium channel inactivation,

A-type potassium activation and A-type potassium inactivation respectively. The code for the model is available at `https://github.com/mkturkcan/FeedbackCircuits/blob/main/feedbackcircuits/NDComponents/AntennalLobe/NoisyConnorStevens.py`.

All synapses between OSNs and LNs, LNs and OSNs, OSNs and PNs, LNs and PNs and, PNs and LNs are modeled as α synapses described by the following equations:

$$\begin{aligned} g^{ji}(t) &= \overline{g}^{ji} s^{ji}(t) \\ \frac{ds^{ji}}{dt}(t) &= h^{ji}(t) 1_{[t \geq 0]}(t) \\ \frac{dh^{ji}}{dt}(t) &= -(a_r^{ji} + a_d^{ji}) h(t) - a_r^{ji} a_d^{ji} s^{ji}(t) + a_r^{ji} a_d^{ji} \sum_k \delta(t - t_k^i), \end{aligned} \tag{S13}$$

where i and j are the indices of the presynaptic and postsynaptic neurons, respectively, $s^{ji}(t)$ and $h^{ji}(t)$ are state variables, and $\overline{g}^{ji}$ is a scaling factor, a_r^{ji} and a_d^{ji} are, respectively, the rise and decay time of the synapse, $1_{[t \geq 0]}(t)$ is the Heaviside function and $\delta(t)$ is the Dirac function. $\delta(t - t_k^i)$ indicates an input spike from the presynaptic neuron at time t_k^i. The code for the model is available at `https://github.com/mkturkcan/FeedbackCircuits/blob/main/feedbackcircuits/NDComponents/AntennalLobe/AlphaSpike.py`.

LN-to-OSN synapses do not provide a current to OSNs. Rather, they act at the presynaptic site of the OSN terminals and modulate the vesicle release (Lazar et al., 2020). Vesicle release in turn determines the postsynaptic current of the OSN-to-PN and OSN-to-LN synapses. The postsynaptic current induced by an OSN-to-PN synapse are given by

$$I_{OSN \to PN} = \gamma \frac{g_{OSN \to PN}}{b + \alpha \sum_i g_{LN_i \to OSN}}, \tag{S14}$$

where γ, b and α are constants. γ approximates the difference between the PN membrane potential and the reversal potential, $g_{OSN \to PN}$ is the conductance of the OSN-to-PN synapse, and $g_{LN_i \to OSN}$ is the conductance of the synapses between the ith LN and an OSN. The aforementioned conductances satisfy the equations in (S13). The postsynaptic current induced by an OSN-to-LN synapse shares the same form as (S14) with PN replaced by LN.

The postsynaptic current induced by an PN-to-LN synapse can be expressed as

$$I_{PN \to LN} = \gamma g_{PN \to LN}, \tag{S15}$$

where γ is a constant, and $g_{PN \to LN}$ is evaluated using Equation S13. The code is available at `https://github.com/mkturkcan/FeedbackCircuits/blob/main/feedbackcircuits/NDComponents/AntennalLobe/OSNAxt2.py`.

According to (Lazar and Yeh, 2020), the OSN odorant transduction process is given by

$$[\mathbf{v}]_{ron} = \textit{Re}\left(\int_{\mathbb{R}} h(t-s)u(s)ds + [\gamma]_{ron}\int_{\mathbb{R}} h(t-s)du(s)\right)$$
$$\begin{bmatrix} \frac{d\mathbf{x}_1}{dt} \\ \frac{d\mathbf{x}_2}{dt} \\ \frac{d\mathbf{x}_3}{dt} \end{bmatrix}_{ron} = \begin{pmatrix} [\mathbf{b}]_{ron}\cdot[\mathbf{v}]_{ron}\cdot(1-[\mathbf{x}_1]_{ron}) - [\mathbf{d}]_{ron}\cdot[\mathbf{x}_1]_{ron} \\ \alpha_2\cdot[\mathbf{x}_1]_{ron}(1-[\mathbf{x}_2]_{ron}) - \beta_2\cdot[\mathbf{x}_2]_{ron} - \kappa\cdot[\mathbf{x}_2]_{ron}^{2/3}\cdot[\mathbf{x}_3]_{ron}^{2/3} \\ \alpha_3\cdot[\mathbf{x}_2]_{ron} - \beta_3\cdot[\mathbf{x}_3]_{ron} \end{pmatrix}$$
$$[\mathbf{I}]_{ron} = \frac{[\mathbf{x}_2]_{ron}^p}{[\mathbf{x}_2]_{ron}^p + c^p}\cdot I_{max},, \tag{S16}$$

where o is the index of a pure odorant, $u(t)$ is the concentration waveform amplitude presented to the antenna, $[\mathbf{I}]_{ron}$ is the transduction current of neuron n expressing receptor r. The biological spike generator of the OSN is modeled as a Connor-Stevens neuron with $[\mathbf{I}]_{ron}$ as the current source.

Note that the differential equation on $[\mathbf{x}_1]_{ron}$ can be rewritten as

$$\frac{1}{[\mathbf{d}]_{ron}}\frac{d[\mathbf{x}_1]_{ron}}{dt} = [\mathbf{a}]_{ron}\cdot[\mathbf{v}]_{ron}\cdot(1-[\mathbf{x}_1]_{ron}) - [\mathbf{x}_1]_{ron}, \tag{S17}$$

where $[\mathbf{a}]_{ron} = [\mathbf{b}]_{ron}/[\mathbf{d}]_{ron}$ is the affinity value of the odorant-receptor pair. The code is available at `https://github.com/FlyBrainLab/EOScircuits/blob/fbl/eoscircuits/antcircuits/NDComponents/OTP.py`.

Table S1. Number of occurrences of port connectivity patterns in the AL. 2nd column: Number of occurrences for all LN innervations in all 51 olfactory glomeruli, where each LN innervation in a glomerulus counts as 1 occurrence. 3rd column: Number of occurrences in the DM4 glomerulus (*i.e.*, the number of LNs that have an port connectivity pattern in DM4). 4th column: Number of occurrences in the DL5 glomerulus (*i.e.*, the number of LNs that have an port connectivity pattern in DL5).

Port Connectivity Pattern	# of Occurrences (all glomeruli)	# of Occurrences (DM4)	# of Occurrences (DL5)
1111	808	21	26
0011	725	13	19
0111	514	14	8
0100	318	8	4
0010	263	1	4
1110	239	6	7
0001	221	4	0
0110	131	7	2
0101	106	4	1
1011	69	0	0
1100	60	0	0
1101	30	1	0
1000	18	1	0
1010	16	0	0
1001	3	0	0

REFERENCES

Connor, J. A. and Stevens, C. F. (1971). Prediction of repetitive firing behaviour from voltage clamp data on an isolated neurone soma. *The Journal of Physiology* 213, 31–53. doi:10.1113/jphysiol.1971.sp009366

Lazar, A. A., Liu, T., and Yeh, C.-H. (2020). An odorant encoding machine for sampling, reconstruction and robust representation of odorant identity. In *ICASSP 2020 - 2020 IEEE International Conference on Acoustics, Speech and Signal Processing (ICASSP)*. 1743–1747. doi:10.1109/ICASSP40776.2020.9054588

Lazar, A. A. and Yeh, C.-H. (2020). A molecular odorant transduction model and the complexity of spatio-temporal encoding in the drosophila antenna. *PLOS Computational Biology* 16, 1–31. doi:10.1371/journal.pcbi.1007751
